# Support for authoritarianism and use of force by and against the federal government in the United States in mid-2025: findings from a nationally representative survey

**DOI:** 10.1186/s40621-026-00689-y

**Published:** 2026-05-26

**Authors:** Garen J. Wintemute, Andrew Crawford, Elizabeth A. Tomsich, Mona A. Wright, Aaron B. Shev, Daniel J. Tancredi, Julia P. Schleimer, Veronica A. Pear, Sonia L. Robinson

**Affiliations:** 1 Department of Emergency Medicine, School of Medicine, UC Davis Medical Center, 2315 Stockton Blvd, Sacramento, CA 95817 USA; 2 Centers for Violence Prevention, University of California Davis, Sacramento, CA USA; 3 Department of Pediatrics, School of Medicine, UC Davis, Sacramento, CA USA

**Keywords:** Political violence, State violence, Authoritarianism, Democracy, Domestic violent extremism, Domestic deployment of the military, Militia movement, Longitudinal survey

## Abstract

**Background:**

During this period of heightened risk for political violence and authoritarian government in the United States (US), there is an urgent need for information on the public’s support for authoritarianism and government-initiated violence and its willingness to engage in pro- or anti-government violence.

**Methods:**

Findings are from Wave 4, conducted May 23-June 13, 2025, of a nationally representative annual longitudinal survey of adult members of the Ipsos KnowledgePanel. Findings are presented for the cohort as a whole and for demographic and political affiliation subgroups. Outcomes are expressed as weighted prevalences and adjusted prevalence differences, with p-values adjusted for the false discovery rate.

**Results:**

The survey completion rate was 89.9%. Of 8248 respondents, 15.0% (95% confidence interval (CI) 13.9%, 16.1%) strongly or very strongly agreed that “having a strong leader for America is more important than having a democracy.” One in 10 (9.6%, 95% CI 8.8%, 10.6%) strongly or very strongly agreed that “the [federal] government should use the military to help enforce its policies in the United States,” and 4.4% (95% CI 3.7%, 5.2%) strongly or very strongly agreed with the use of “private armed militia groups” for that purpose. Between 5% and 7% of respondents strongly or very strongly agreed that the federal government should arrest its critics, and about 2% were very or completely willing to personally engage in violence in support of (or opposition to) “government policies or the President” and against individual opponents (or supporters) of the government. Younger respondents tended to be less supportive of democracy as a form of government, more supportive of statements consistent with authoritarianism, and more willing to engage in pro- or anti-government violence. Compared with strong Democrats, “Make America Great Again” (MAGA) Republicans were more supportive of statements consistent with authoritarianism and the current federal government’s use of the military and private militia groups but were not more willing personally to engage in violence.

**Conclusions:**

In 2025, substantial minorities of US adults endorsed statements consistent with authoritarianism. Smaller minorities endorsed and were willing to engage in pro- or anti-government violence. These findings call for sustained, concerted action to avert widespread political violence and preserve democracy in the United States.

**Supplementary Information:**

The online version contains supplementary material available at 10.1186/s40621-026-00689-y.

## Background

For nearly a decade, researchers and policy experts have warned that the United States (US) is at risk for political violence [1–7]. Evidence from survey research has substantiated that warning: in 2023 and 2024, while most Americans rejected political violence, about 25% of them considered it justified under specific conditions [8]. Support for political violence increased further in 2025 [9]. This occurred in association with the ascension into power of a federal administration led by a president who has endorsed violence by the government, including the military, against civilians [10] and whose public statements have been linked with political violence in several studies [11–15].

Concern has also grown among experts and policy makers that democracy itself is at risk in the US and that the current US administration is seeking to establish an authoritarian form of government [16–19]. Research on authoritarianism has grown substantially in recent years; a Web of Science topic search on [“authoritarianism United States”], conducted April 14, 2026, yielded 20 or fewer publications per year until 2016 but nearly 90 in 2025.

There exists a “shared constellation of personality traits, cognitive features, beliefs, and motivational values that might be considered the ‘heart’ of authoritarianism[: ]…preference for social uniformity, prejudice toward different others, willingness to wield group authority to coerce behavior, cognitive rigidity, aggression and punitiveness toward perceived enemies, outsized concern for hierarchy, and moral absolutism” [20]. Long associated with the political far right [21–24], authoritarianism has more recently been associated with the political far left as well [20, 25, 26]. Distinct right-wing and left-wing authoritarian phenotypes (including demographics, personality traits, and attitudes and beliefs) have made it important to distinguish between authoritarianism itself and political ideology [20, 27, 28].

In the US, political violence has for more than a decade been associated predominantly with the political far right [1, 3, 7, 29–32]. Violence from the right fell precipitously in 2025, however [33]; as the leader of the Proud Boys commented to the New York Times that August, “Honestly, what have we got to complain about?” [34]. As a result, instances of violence arising from the political left, without increasing beyond the level set during the prior decade, exceeded those arising from the right for the first time in more than 30 years [33].

In mid-2022, we initiated an annual, nationally representative longitudinal survey in the US [8, 9, 35] that seeks to establish the prevalence and determinants of support for and willingness to commit political violence and to track the prevalences over time. Since its inception, the survey has also gathered information on respondents’ support for democracy as a form of government and for statements consistent with authoritarianism, their political party affiliation, and their political ideology [8, 9, 35–37].

Over the multi-year course of the survey, we have found that some subsets of the population are more supportive of political violence than others: Republicans more than Democrats, for example, and MAGA Republicans more than other Republicans [36, 37]. Men more than women [35]. Persons who endorse expressions of racism, hostile sexism, homophobia, transphobia, xenophobia, Islamophobia, and antisemitism [38]. Supporters of the great replacement theory, Christian nationalism, QAnon, the white supremacy movement, the militia movement, and organizations such as the Proud Boys and Oath Keepers [39, 40]. Firearm owners—but only to a small degree, unless they owned assault-type rifles, had purchased firearms recently, or habitually carried loaded firearms in public [41]. In these studies, support for political violence and agreement with expressions authoritarianism have varied in parallel.

We designed the 2025 survey in response to events occurring in the US after the 2024 survey had been in the field and to the heightened concerns for political violence and authoritarian government described earlier. We repeated existing questionnaire items on those topics and added new items designed to assess respondents’ views on violence initiated by the current federal government and respondents’ personal willingness to engage in violence in support of or in opposition to current federal government policies. To our knowledge, these topics have not been studied previously; this study thus extends prior knowledge. The aim of the study is to provide evidence that will strengthen efforts in the US to prevent political violence and the rise of authoritarianism.

## Methods

Methods for Wave 4 of this longitudinal survey closely followed those for Waves 1–3 [8, 35, 42]. Wave 4 was designed by the authors and administered online in English and Spanish from May 23 to June 13, 2025 by the survey research firm Ipsos [43]. The study was reviewed by the University of California Davis Institutional Review Board (IRB; protocol 187125: exempt from full review, category 2, survey research). The IRB waived a requirement for written or verbal consent. Before participants accessed the questionnaire, they were provided informed consent language that concluded, “[by] continuing, you are agreeing to participate in this study.” The study is reported following American Association for Public Opinion Research guidelines [44].

### Participants

Participants for 2022’s Wave 1 were drawn from the Ipsos KnowledgePanel, an online research panel that has been widely used in population-based research on violence and firearm ownership [45–49]. To establish a nationally representative panel, KnowledgePanel members are recruited on an ongoing basis through address-based probability sampling using data from the US Postal Service’s Delivery Sequence File [50, 51]. Recruitment into KnowledgePanel involves repeated contact attempts, if necessary, by mail and telephone. Recruited adults in households without internet access are provided a web-enabled device and free internet service, and a modest, primarily points-based incentive program seeks to encourage participation and promote participants’ retention in KnowledgePanel over time [50, 51].

A probability-proportional-to-size procedure was used to select a study-specific sample for Wave 1. All panel members who were aged 18 years and older were eligible for selection. Invitations were sent by e-mail; automatic reminders were delivered to non-respondents by e-mail and telephone beginning 3 days later [50, 51].

A detailed participation history for Waves 1–4 has been reported previously [8, 9]. For 2025’s Wave 4, invitations to participate were sent to the 9,179 Wave 1 respondents who remained active members of KnowledgePanel on Wave 4’s launch date (see Supplement, Additional file 1, Figure S1).

A final Wave 4 survey weight variable for longitudinal analyses was provided by Ipsos. It adjusted for the initial probability of selection into KnowledgePanel, for survey-specific nonresponse and over- or under-coverage, and for differential attrition using design weights with post-stratification raking ratio adjustments. As with prior samples, the weighted 2025 sample is designed to be statistically representative of the noninstitutionalized adult population of the US as reflected in the 2021 March supplement of the Current Population Survey [50, 51].

### Measures

Sociodemographic data were collected by Ipsos from profiles created and maintained by KnowledgePanel members. Questions addressing respondents’ support for democracy as a form of government and for statements consistent with authoritarianism, their political party, and their “Make America Great Again” (MAGA) movement affiliation were repeated from prior waves. Questions on actions the federal government might take to enforce its policies in 2025 and on respondents’ willingness to commit violence in support of or opposition to current federal government policies had not been asked previously.

The questions on democracy and authoritarianism and on actions the government might take were placed at the beginning of the 2025 questionnaire. The 3 statements consistent with authoritarianism were designed to be independent of ideology. Respondents were asked about their agreement with these statements: “Having a strong leader for America is more important than having a democracy,” and “We should suspend Congress for a few years so a strong leader can clean up the mess made by politicians in Washington.” They were also asked to choose “Which is more important to you…” (a) “Having election outcomes determined democratically,” or (b) “Having political leaders I can trust to look out for my values and interests.” The first item was developed by the authors; the second [52] and third [53] had been developed by other investigators.

They were then asked about their agreement with “actions the federal government might take this year.” This series began with “the government should use the military” (and, separately, “private armed militia groups”) “to help enforce its policies in the United States.” It continued with 3 items on whether “the government should arrest” critics of “its policies or the President”: “people who speak out,” “people who join demonstrations,” and “reporters and journalists whose stories are critical.”

As in prior waves, respondents were presented items later in the questionnaire about the extent to which they considered political violence to be justified “in general” and then about justification for its use to advance a series of specified political objectives. Violence was again represented in the questionnaire by the phrase “force or violence,” defined as “physical force strong enough that it could cause pain or injury to a person.” We described political violence as “force or violence to advance an important political objective that you support.”

Respondents who considered political violence to be sometimes, usually, or always justified to advance at least 1 objective were presented questions about their personal willingness to commit political violence, including the new questions on violence to support or oppose the government that we assess here. The first of these asked whether respondents were generally willing to “personally use force or violence” against the 3 types of critics described above of “government policies or the President.” The second asked about willingness to commit violence against 3 comparable types of government supporters. Those two items were not specific to the present time or the current government. Finally, respondents were asked about their willingness “in general” to “use force or violence *this year*” to “support” (or, in a separate item, “oppose”) “the government’s enforcement of its policies.”

All questions that provided data for this analysis are in the Supplement (see Supplement, Additional file 1).

### Implementation

Ipsos translated the questionnaire into Spanish. Twenty-three KnowledgePanel members participated in a pretest of the English language version that was administered May 16–20, 2025.

Respondents were randomized 1:1 to receive response options in order from either negative to positive valence (example: from ‘do not agree’ to ‘strongly agree’) or the reverse throughout the questionnaire. Where a question presented multiple statements for respondents to consider, the order in which those statements were presented was randomized unless ordering was necessary. Logic-driving questions (those to which responses might invoke a skip pattern) included non-response prompts.

We employed unipolar response arrays without a neutral midpoint (for example, do not agree, somewhat agree, strongly agree, very strongly agree). The literature is not in agreement on whether such midpoints should be included [54, 55]. We were persuaded by the studies reviewed by Chyung et al. [54], which suggest that such midpoints allow respondents to choose “a minimally acceptable response as soon as it is found, instead of putting effort to find an optimal response,” a behavior known as satisficing. According to those authors, satisficing is particularly common when respondents are uncomfortable with the topics of the survey or under social desirability pressures; both conditions apply here.

### Statistical analysis

Most survey items had ordinal response options. We combined the response options usually justified and always justified, very willing and completely willing, and very likely and extremely likely for the analysis. To generate prevalence estimates, we calculated weighted percentages and 95% confidence intervals (CI) using Complex Samples Frequencies in IBM SPSS Statistics, version 30 (IBM Corp., Armonk, NY) and PROC SURVEYFREQ in SAS version 9.4 (SAS Institute, Inc., Cary, NC).

Estimated counts of adults in the US were generated by simple extrapolation from the population-representative results, multiplying weighted percentages and their confidence intervals from our sample by the estimated adult population of the US as of July 1, 2024 (266.98 million persons) [56].

Comparisons based on sociodemographic characteristics were qualitative and were not adjusted; a difference between 2 groups was determined to exist when there was no overlap between 95% confidence intervals for their estimated prevalences of an outcome of interest.

To assess differences by political party and MAGA affiliations, we computed adjusted prevalence differences (aPDs) and their 95% CIs. We defined outcomes dichotomously (example: usually/always justified and never/sometimes justified) and used linear regression via PROC SURVEYREG in SAS, employing robust standard errors to correct for design effects and heteroskedasticity in binary outcomes and weights to account for the complex survey design. We defined strong Democrats as the reference group. The model included respondent age, gender, race and ethnicity, education, income, Census division, marital status, homeownership, rurality, firearm ownership, alcohol consumption, military service, and history of non-traffic arrest (see Supplement, Additional file 1 for variable specifications). We expressed aPDs and their CIs in absolute percentage points (pp).

We controlled for multiple comparisons by bounding the false discovery rate (FDR) using the Benjamini-Yekutieli method [57], a conservative approach that is appropriate when associations between exposures and outcomes are not hypothesized to be all positive or all negative. The resulting p-values are known as FDR-adjusted (or FDR-corrected) p-values or as q-values [58]; we employ the latter term here. For a given value of the false discovery rate, Q (Q = 0.05 was chosen here), the q-values are chosen so that, of comparisons that reject the null hypothesis (decided by q < α), the expected proportion of these comparisons that incorrectly reject the null hypothesis is at most Q.

## Results

Invitations were sent to 9179 persons, of whom 8248 responded (response rate 89.9%). The median survey completion time was 23.6 min (interquartile range, 17.2 min). One outcome item had a non-response percentage above 3.0% (see Supplement, Additional file 1); for all others, non-response ranged from 0.7% to 1.7%.

After weighting, half of the respondents (50.7%, 95% CI 49.2%, 52.2%) were female; 62.7% (95% CI 61.2%, 64.3%) were white, non-Hispanic (see Supplement, Additional file 1, Table S1). The weighted mean (SD) respondent age was 48.5 (24.9) years. Nonrespondents were less frequently male and white, non-Hispanic (see Supplement, Additional file 1, Table S2).

### Cohort-wide findings

#### Democracy and authoritarianism

The vast majority of respondents (88.0%, 95% CI 86.8%, 89.0%; *N* = 234.9 million, 95% CI 231.8 million, 237.7 million) considered it very or extremely important that the US remain a democracy (Table 1), and most (61.1%, 95% CI 59.7%, 62.6%; *N* = 163.2 million, 95% CI 159.3 million, 167.1 million) reported believing that there is “a serious threat to our democracy.” More than two thirds (68.8%, 95% CI 67.3%, 70.2%; *N* = 187.3 million, 95% CI 179.8 million, 187.5 million) strongly or very strongly agreed that “democracy is the best form of government.”

**Table 1:** Views on democracy and authoritarianism

The United States as you see it now, in 2025	Respondents (*n* = 8248)		Estimated *N* of adults in US*
	Unweighted *n*	Weighted % (95% CI)	*N* (95% CI) (in millions)
How important do you think it is for the United States to remain a democracy?			
Not important	192	3.3 (2.7, 3.9)	8.8 (7.3, 10.5)
Somewhat important	363	7.1 (6.3, 8.1)	19.1 (16.8, 21.6)
Very or extremely important	7594	88.0 (86.8, 89.0)	234.9 (231.8, 237.7)
Non-response	99	1.6 (1.2, 2.0)	4.2 (3.3, 5.5)
When thinking about democracy in the United States these days, do you believe…			
There is a serious threat to our democracy	5236	61.1 (59.7, 62.6)	163.2 (159.3, 167.1)
There may be a threat to our democracy, but it is not serious	1878	25.0 (23.7, 26.4)	66.8 (63.3, 70.4)
There is no threat to our democracy	999	11.2 (10.3, 12.1)	29.8 (27.4, 32.3)
Non-response	135	2.7 (2.2, 3.3)	7.2 (5.8, 8.9)
How much do you agree or disagree with the following statements about democracy in the United States?			
Democracy is the best form of government			
Do not agree	468	6.9 (6.1, 7.8)	18.4 (16.3, 20.7)
Somewhat agree	1408	21.9 (20.6, 23.2)	58.5 (55.1, 62.1)
Strongly or very strongly agree	6252	68.8 (67.3, 70.2)	183.7 (179.8, 187.5)
Non-response	120	2.4 (1.9, 3.0)	6.4 (5.1, 8.0)
Having a strong leader for America is more important than having a democracy			
Do not agree	5709	64.9 (63.5, 66.4)	173.4 (169.5, 177.2)
Somewhat agree	1279	17.5 (16.4, 18.7)	46.8 (43.8, 50.0)
Strongly or very strongly agree	1120	15.0 (13.9, 16.1)	39.9 (37.1, 42.9)
Non-response	140	2.6 (2.1, 3.2)	6.9 (5.6, 8.5)
We should suspend Congress for a few years so a strong leader can clean up the mess made by politicians in Washington.			
Do not agree	6193	69.7 (68.3, 71.1)	186.1 (182.3, 189.8)
Somewhat agree	1094	16.0 (14.8, 17.1)	42.6 (39.6, 45.7)
Strongly or very strongly agree	827	11.6 (10.6, 12.6)	30.9 (28.4, 33.6)
Non-response	134	2.7 (2.2, 3.4)	7.3 (6.0, 9.0)
Which is more important to you…			
Having election outcomes determined democratically	5458	60.4 (58.9, 61.8)	161.2 (157.2, 165.1)
Having political leaders I can trust to look out for my values and interests	2496	33.7 (32.3, 35.1)	90.0 (86.3, 93.8)
Non-response	294	5.9 (5.1, 6.8)	15.8 (13.7, 18.2)

* Sample population extrapolations are based on the estimated US adult (age ≥ 18 years) population, *N* = 266,978,268 as of July 1, 2024 [42]

However, 15.0% (95% CI 13.9%, 16.1%) of respondents (*N* = 39.9 million, 95% CI 37.1 million, 42.9 million) strongly or very strongly agreed that “having a strong leader for America is more important than having a democracy,” and 11.6% (95% CI 10.6%, 12.6%; *N* = 30.9 million, 95% CI 28.4 million, 33.6 million) strongly or very strongly agreed with a proposal to “suspend Congress for a few years so a strong leader can clean up the mess made by politicians in Washington” (Table 1). One third of respondents (33.7%, 95% CI 32.3%, 35.1%; *N* = 90.0 million, 95% CI 86.3 million, 93.8 million) believed that “having political leaders I can trust to look out for my values and interests” was more important than “having election outcomes determined democratically.” Several of these items had been presented in prior waves of the survey, and findings for 2025 differed little from those for 2022–2024 (see Supplement, Additional file 1, Table S3).

#### Support for government-initiated violence

Just under 10% of respondents (9.6%, 95% CI 8.8%, 10.6%; *N* = 25.8 million, 95% CI 23.5 million, 28.3 million) strongly or very strongly agreed that among the “actions the federal government might take this year” (in 2025), it “should use the military to help enforce its policies in the United States,” and 4.4% (95% CI 3.7%, 5.2%; *N* = 11.7 million, 95% CI 10.0 million, 13.8 million) strongly or very strongly agreed that the federal government “should use private armed militia groups” for that purpose (Table 2). About 6% of respondents strongly or very strongly agreed that the federal government should arrest ordinary people and reporters or journalists who publicly oppose or criticize “its policies or the President” (Table 2).

**Table 2:** Support for violence initiated by the federal government

Actions the federal government might take this year	Prevalence 2025 (*n* = 8248)		Estimated *N* of adults in US*
	Unweighted *n*	Weighted % (95% CI)	*N* (95% CI) (in millions)
How much do you agree or disagree with the following statements?			
The government should use the military to help enforce its policies in the United States.			
Do not agree	5496	65.4 (64.0, 66.8)	174.7 (170.9, 178.4)
Somewhat agree	1866	22.6 (21.3, 23.8)	60.2 (57.0, 63.6)
Strongly or very strongly agree	761	9.6 (8.8, 10.6)	25.8 (23.5, 28.3)
Non-response	125	2.4 (1.9, 2.9)	6.3 (5.1, 7.9)
The government should use private armed militia groups to help enforce its policies in the United States.			
Do not agree	7248	83.7 (82.5, 84.9)	223.6 (220.3, 226.7)
Somewhat agree	612	9.3 (8.4, 10.3)	24.9 (22.5, 27.5)
Strongly or very strongly agree	261	4.4 (3.7, 5.2)	11.7 (10.0, 13.8)
Non-response	127	2.5 (2.0, 3.1)	6.8 (5.5, 8.3)
And how much do you agree or disagree with these statements? The government should arrest…			
People who speak out against its policies or the President			
Do not agree	7137	83.0 (81.8, 84.2)	221.6 (218.4, 224.7)
Somewhat agree	608	9.0 (8.1, 9.9)	23.9 (21.6, 26.5)
Strongly or very strongly agree	371	5.5 (4.8, 6.2)	14.6 (12.8, 16.6)
Non-response	132	2.5 (2.1, 3.2)	6.8 (5.5, 8.4)
People who join demonstrations against its policies or the President			
Do not agree	6738	78.6 (77.3, 79.9)	209.9 (206.5, 213.3)
Somewhat agree	929	12.6 (11.6, 13.7)	33.7 (31.0, 36.5)
Strongly or very strongly agree	448	6.2 (5.5, 7.0)	16.7 (14.8, 18.8)
Non-response	133	2.5 (2.0, 3.1)	6.7 (5.4, 8.2)
Reporters and journalists whose stories are critical of its policies or the President			
Do not agree	6992	80.8 (79.5, 82.0)	215.6 (212.2, 218.9)
Somewhat agree	694	10.4 (9.5, 11.4)	27.8 (25.3, 30.5)
Strongly or very strongly agree	437	6.2 (5.5, 7.0)	16.6 (14.7, 18.7)
Non-response	125	2.6 (2.1, 3.2)	7.0 (5.6, 8.6)

* Sample population extrapolations are based on the estimated US adult (age ≥ 18 years) population, *N* = 266,978,268 as of July 1, 2024 [42]

#### Engagement in violence

Being very or completely willing personally to commit violence to support or to oppose “the government’s enforcement of its policies” was uncommon in 2025 (Table 3): to support the government, 2.1% (95% CI 1.6%, 2.6%; *N* = 5.5 million, 95% CI 4.3 million, 7.0 million); to oppose it, 2.7% (95% CI 2.2%, 3.3%; *N* = 7.1 million, 95% CI 5.8 million, 8.8 million). These small proportions were not statistically significantly different. Similarly, about 2% of respondents were very or completely willing to use force themselves against ordinary people or against reporters or journalists who either opposed or supported the government (Table 3).

**Table 3:** Personal willingness to engage in violence in support of or opposition to the federal government

How willing would *you personally* be?	Prevalence (*n* = 8248)		Estimated *N* of adults in US*
	Unweighted *n*	Weighted % (95% CI)	*N* (95% CI) (in millions)
In general, in a situation where you think force or violence is justified to advance an important political objective, how willing would *you personally* be to use force or violence *this year*…			
To *support* the government’s enforcement of its policies			
Not asked the question†	1653	22.3 (21.1, 23.6)	59.6 (56.4, 63.0)
Not willing	5747	67.8 (66.3, 69.2)	180.9 (177.1, 184.6)
Somewhat willing	588	6.5 (5.8, 7.3)	17.4 (15.5, 19.5)
Very or completely willing	125	2.1 (1.6, 2.6)	5.5 (4.3, 7.0)
Non-response	78	1.3 (1.0, 1.8)	3.5 (2.6, 4.7)
To *oppose* the government’s enforcement of its policies			
Not asked the question†	1653	22.3 (21.1, 23.6)	59.6 (56.4, 63.0)
Not willing	5516	63.8 (62.3, 65.2)	170.3 (166.4, 174.1)
Somewhat willing	800	9.8 (9.0, 10.8)	26.3 (23.9, 28.8)
Very or completely willing	144	2.7 (2.2, 3.3)	7.1 (5.8, 8.8)
Non-response	78	1.4 (1.0, 1.8)	3.6 (2.7, 4.8)
In a situation where you think force or violence is justified to advance an important political objective, how willing would *you personally* be to use force or violence against a person *because* they are…			
A person who speaks out against government policies or the President			
Not asked the question†	1653	22.3 (21.1, 23.6)	59.6 (56.4, 63.0)
Not willing	6133	71.1 (69.7, 72.4)	189.7 (186.0, 193.4)
Somewhat willing	239	3.4 (2.8, 4.0)	9.0 (7.6, 10.7)
Very or completely willing	105	2.1 (1.7, 2.7)	5.6 (4.4, 7.2)
Non-response	61	1.1 (0.8, 1.5)	2.9 (2.1, 4.1)
A person who joins demonstrations against government policies or the President			
Not asked the question†	1653	22.3 (21.1, 23.6)	59.6 (56.4, 63.0)
Not willing	6032	70.2 (68.7, 71.5)	187.3 (183.5, 190.9)
Somewhat willing	333	4.4 (3.8, 5.1)	11.7 (10.0, 13.6)
Very or completely willing	104	2.0 (1.5, 2.5)	5.2 (4.1, 6.6)
Non-response	69	1.2 (0.9, 1.6)	3.2 (2.3, 4.3)
A reporter or journalist whose stories are critical of government policies or the President			
Not asked the question†	1653	22.3 (21.1, 23.6)	59.6 (56.4, 63.0)
Not willing	6129	71.2 (69.8, 72.6)	190.1 (186.4, 193.7)
Somewhat willing	238	3.4 (2.8, 4.0)	9.0 (7.6, 10.6)
Very or completely willing	104	1.9 (1.5, 2.5)	5.1 (4.0, 6.6)
Non-response	67	1.2 (0.9, 1.6)	3.2 (2.3, 4.3)
This question turns things around: in a situation where you think force or violence is justified to advance an important political objective, how willing would *you personally* be to use force or violence against a person *because* they are…			
A person who speaks out *in favor* of government policies or the President			
Not asked the question†	1653	22.3 (21.1, 23.6)	59.6 (56.4, 63.0)
Not willing	6097	70.3 (68.9, 71.7)	187.7 (183.9, 191.3)
Somewhat willing	261	4.1 (3.5, 4.8)	11.0 (9.4, 12.9)
Very or completely willing	107	1.9 (1.5, 2.5)	5.1 (4.0, 6.6)
Non-response	73	1.3 (1.0, 1.7)	3.5 (2.6, 4.6)
A person who joins demonstrations *in favor* of government policies or the President			
Not asked the question†	1653	22.3 (21.1, 23.6)	59.6 (56.4, 63.0)
Not willing	6087	70.1 (68.7, 71.5)	187.3 (183.5, 190.9)
Somewhat willing	275	4.4 (3.8, 5.1)	11.8 (10.1, 13.7)
Very or completely willing	99	1.8 (1.4, 2.3)	4.8 (3.7, 6.2)
Non-response	77	1.3 (1.0, 1.7)	3.5 (2.6, 4.7)
A reporter or journalist whose stories *support* government policies or the President			
Not asked the question†	1653	22.3 (21.1, 23.6)	59.6 (56.4, 63.0)
Not willing	6087	70.1 (68.7, 71.5)	187.3 (183.5, 190.9)
Somewhat willing	275	4.4 (3.8, 5.1)	11.8 (10.1, 13.7)
Very or completely willing	99	1.8 (1.4, 2.3)	4.8 (3.7, 6.2)
Non-response	77	1.3 (1.0, 1.7)	3.5 (2.6, 4.7)

* Sample population extrapolations are based on the estimated US adult (age ≥ 18 years) population, *N* = 266,978,268 as of July 1, 2024 [42]

† Respondents (*n* = 1653) who were not asked the question had indicated violence was never justified to achieve any political objective; an additional 57 respondents who did not answer the question “In general.to advance an important political objective that you support” were not asked the question

### Comparisons between subgroups

#### Demographic characteristics

Younger respondents were less likely to endorse statements of support for democracy as a form of government, and white, non-Hispanic respondents were somewhat less likely than those in most other racial and ethnic groups to endorse expressions of authoritarianism (see Supplement, Additional file 1, Table S4). Women were less likely than men to endorse democracy and more likely to endorse authoritarianism.

Younger respondents were also more likely to endorse the government’s use of the military and private militia groups to enforce its policies in the US, but they were more likely to support the arrest of government critics in only 1 of 3 instances (“people who speak out”) (see Supplement, Additional file 1, Table S5). Gender differences in strong or very strong agreement on these items were very small (women more in agreement than men) where they existed. White, non-Hispanic respondents tended to have lower levels of agreement with these items than did respondents of other races and ethnicities, but differences were again small.

Younger respondents were also more likely to be very or extremely willing to engage in violence, whether in support of or opposition to the government (see Supplement, Additional file 1, Table S6). Gender differences were very small, where they existed. White, non-Hispanic respondents were generally least willing to engage in violence.

#### Political party and MAGA affiliation

Categorization by political party and MAGA affiliation yielded 8 subgroups (see Supplement, Additional file 1, Table S7). For simplicity, the following text presents results for MAGA Republicans and strong Democrats; the tables include all subgroups.

MAGA Republicans were less likely than strong Democrats to endorse statements of support for democracy and more likely to endorse statements consistent with authoritarianism (See Supplement, Additional File 1, Table S7). For example, MAGA Republicans were less likely to strongly or very strongly agree that “democracy is the best form of government” (MAGA Republicans 69.2%, 95% CI 65.8%, 72.4%; strong Democrats 83.8%, 95% CI 80.8%, 86.5%; aPD − 17.66pp, 95% CI -22.08pp, -13.24pp; q < 0.0001) and more likely to strongly or very strongly agree that “having a strong leader for America is more important than having a democracy” (MAGA Republicans 32.1%, 95% CI 28.9%, 35.4%; strong Democrats 7.5%, 95% CI 5.8%, 9.7%; aPD 26.38pp, 95% CI 22.24pp, 30.52pp; q < 0.0001).

Findings on the domestic use of force by the federal government were mixed (see Supplement, Additional file 1, Table S8). MAGA Republicans were more likely than strong Democrats to strongly or very strongly agree that the federal government “should use the military to help enforce its policies in the United States” (MAGA Republicans 23.6%, 95% CI 20.9%, 26.7%; strong Democrats 4.1%, 95% CI 2.7%, 6.1%; aPD 22.16pp, 95% CI 18.51pp, 25.80pp; q < 0.0001) and, to a lesser extent, to endorse the government’s use of “private armed militia groups” (MAGA Republicans 9.6%, 95% CI 7.6%, 12.2%; strong Democrats 1.9%, 95% CI 1.0%, 3.7%; aPD 9.60pp, 95% CI 6.82pp, 13.38pp; q < 0.0001). They were not more likely to endorse the arrest of government critics, whether in the general public or the media.

MAGA Republicans were not more willing than strong Democrats to engage in political violence (see Supplement, Additional file 1, Table S9), whether “to support the government’s enforcement of its policies” (very or completely willing: MAGA Republicans 2.4%, 95% CI 1.5%, 3.8%; strong Democrats 1.3%, 95% CI 0.6%, 1.9%; aPD 2.02pp, 95% CI 0.41pp, 3.63pp; q = 0.18) or against the 3 categories of critics of the government.

Personal willingness to engage in violence was uncommon across the entire range of political party/MAGA affiliations—the prevalence of being very or completely willing did not exceed 5% on any measure—with 1 repeated exception. Among the small group of non-Republican MAGA supporters (*n* = 194, 2.4% of respondents), very/complete willingness was 17.0% (95% CI 9.8%, 27.7%) for violence to support the government—and 15.6% (95% CI 8.8%, 26.0%) to oppose it—and ranged generally from 10% to 15% for violence against 6 categories of government critics and supporters.

## Discussion

Our findings regarding support for democratic government in the US in 2025 are mixed. One third of our respondents—and an estimated 81–92 million adults in the US—agree at least somewhat that “having a strong leader” is more important than “having a democracy” and consider having leaders who align with their values and interests to be more important than having democratic elections. More than a quarter agree at least somewhat with suspending Congress in favor of government by that strong leader. Arguably, these findings justify the position of the nearly two thirds of our respondents who believe that democracy in the US faces “a serious threat.”

It is somewhat reassuring that across multiple waves of the survey we have found no increase over time in agreement with statements consistent with authoritarianism [8, 9, 35]. A surge in such support, at least as measured by the survey, did not precede (and therefore presumably did not influence) the 2024 elections. Nor were those elections, which installed an administration that many experts view as authoritarian [16–19], followed in 2025 by greater support for those statements. But while support for expressions of authoritarianism remained a minority view across all 4 years of the survey, that minority arguably provides a climate of acceptance that enables those seeking to transition the US to a non-democratic form of government. Three examples follow.

First, a third of respondents—by extrapolation, between 80 and 92 million adults in the US—at least somewhat agreed with the current federal government’s use of the military against civilians within the US. This began in 2025 with the deployment of the Marines and/or National Guard in Los Angeles, Portland, Chicago, Memphis, Washington, D.C, and elsewhere [59–61]. The administration is creating a National Guard Domestic Civil Disturbance Quick Reaction Force of 600 troops who would be ready at all times for immediate deployment to quell civil disturbances anywhere in the US [62] and has ordered additional training in civil unrest for the existing National Guard Reaction Force—up to 23,500 troops from all 50 states [63]. Two Army infantry battalions and hundreds of military police officers were temporarily mobilized in January 2026, in conjunction with President Trump’s threat to invoke the Insurrection Act [64, 65].

Second, an estimated 32–41 million US adults at least somewhat support the government’s use of private armed militia groups to enforce its policies. This may also have occurred, in effect, by recruitment of militia members into federal law enforcement, particularly the Immigration and Customs Enforcement (ICE) agency. Militia groups have actively sought involvement in mass deportation efforts [66], and there has been concern since 2024 that private militia members might be recruited by the current administration to enforce its policy agenda or suppress dissent [67, 68]. The White House proclamation launching mass deportation [69] required the Department of Homeland Security to hire at least 20,000 additional enforcement officers. H.R. 1 [70], which became law on July 4, 2025, provided the funding. ICE has since launched a $100 million “wartime recruitment” effort [71], with lowered recruiting and training standards [72, 73], that targets firearm enthusiasts and nativists and appears to celebrate violence [71].

Research findings from this cohort suggest that deployment of private militia members and other right-wing extremists as part of federal law enforcement would likely increase the risk of government-initiated violence in the US. In our 2022 survey, respondents who approved of the militia movement and organizations such as the Proud Boys and Oath Keepers were far more willing than others to commit political violence, including homicide, to advance political objectives [40].

Third, 15% to 20% of respondents—an estimated 34–50 million US adults—at least somewhat support expansion of the federal government’s use of force beyond the implementation of its policies to the suppression of dissent. Here again the government has already proceeded, as in its detention of Senator Alex Padilla at a Department of Homeland Security press conference [74] and arrests and other legal actions targeting elected officials and protesters.

The administration has also laid the groundwork for much more extensive efforts of this sort. In September, 2025, the White House issued “Countering Domestic Terrorism and Organized Political Violence” [75], a national security memorandum alleging a threat to the nation from the political left. It ordered the National Joint Terrorism Task Force to “investigate, prosecute, and disrupt” this threat. Attorney General Bondi provided the specifics in December [76]. According to her memorandum to federal prosecutors and law enforcement agencies, “threat” includes anyone contemplating violence whose views include “opposition to law and immigration enforcement;… adherence to radical gender ideology, anti-Americanism, anti-capitalism, or anti-Christianity;…[and] hostility towards traditional views on family, religion, and morality.” She directed the FBI to provide cash rewards for information leading to arrests. As of spring 2026, the extent to which these directives will be translated into action remains unclear.

Some of the findings of this study provide grounds for hope and guidance for intervention. Personal willingness to commit political violence was uncommon, as it has been in other studies from this survey [8, 9]. More broadly, here as previously, the majority of those who support political violence in principle are unwilling to commit violence themselves. In 2024, we also found that nearly half of the small minority of respondents who fully expected to participate in political violence were open to change in response to input from their families and others [77]. That finding involved a specific circumstance, however—service as a combatant if civil war broke out—and may not generalize to pro- or anti-government violence considered broadly.

In any case, these low prevalences of willingness to commit violence suggest opportunities to prevent that violence. The far larger numbers of Americans who reject political violence can create a population-level climate of non-acceptance that would provide a deterrent, can seek to dissuade family members and others who are willing to commit violence, and can contact trusted authorities in cases where violence appears imminent. This will not be enough if the current administration is committed to the use of violence, if necessary, to enforce its policies. If they are not, however, widespread public rejection of political violence might help prevent that commitment from forming.

The subgroup findings were as expected. As compared with older respondents, those less than 25 years old were strikingly less supportive of democracy, more supportive of authoritarianism and authoritarian violence, and more willing to engage in pro- or anti-government violence. Public opinion polls have recently reported similar findings [78, 79]. The implications for the future of the United States are ominous, and further effort to understand and modify these views is urgently needed.

Findings on political party/MAGA affiliation are similar to those from prior studies from this survey [36, 37], with MAGA adherents more supportive of statements consistent with authoritarianism and authoritarian violence (a majority at least somewhat supported the current government’s use of the military to enforce its policies) but not more willing to engage in that violence themselves. As we have noted elsewhere in greater detail [37], this raises the possibility of some MAGA adherents serving as credible messengers to others who are contemplating violence. It is therefore particularly important for members of this population who oppose violence to make their opposition known.

Also noted previously [37] is the existence of a small group of non-Republican MAGA supporters, demographically distinct from MAGA Republicans and much more supportive than other groups of violence either in support of or opposition to the government. That finding suggests their support for violence is independent of political ideology, which is a particular concern given the emergence of nihilistic violence [80, 81]. Further research on this group should be a priority.

### Limitations

The findings are cross-sectional and, weighting notwithstanding, are subject to selection and information biases due to nonresponse, social desirability, and other factors. In particular, to the extent that respondents viewed personal willingness to commit violence as stigmatized behavior, our results may reflect under-reporting. All such shortcomings can be magnified by extrapolation to the population as a whole. Respondents were 18 or more years years old as of recruitment in 2022 but 21 or more years old as of 2025. Given our findings on age, the lack of data on persons ages 18–20 in 2025 is unfortunate. The Benjamini-Yekutieli (B-Y) method of controlling for multiple comparisons [57] is more conservative than the Benjamini-Hochberg (B-H) method [82] used in earlier studies from this survey [36, 37]—roughly equivalent to using the B-H method with the threshold for statistical significance set at < 0.01. Comparisons to findings from those earlier studies [36, 37] should be based on the aPDs, not the q-values.

We continue to believe that the most plausible interpretation of the items forcing a choice between a ‘strong leader’ and ‘a democracy’ and endorsing the suspension of Congress in favor of leadership by a single individual is that these items measure support for authoritarianism. We did not validate that interpretation, however, and alternative interpretations are possible.

The survey was in the field in June, 2025, when a widely publicized arson assault on a Jewish community event occurred in Colorado [83] and when National Guard troops (and subsequently Marines) were deployed to participate in deportation efforts in Los Angeles [59, 60].

## Conclusion

Findings from this large, nationally representative survey demonstrate that support for statements consistent with authoritarianism in the US, at least as measured here, is surprisingly common but also a minority view, with a stable prevalence through 2025. Substantial minorities of Americans support use of the military by the federal government to enforce its policies and suppress dissent, and small and roughly equal minorities are willing to commit violence in support of or opposition to the federal government and its opponents or supporters. Those who reject violence should join in efforts to prevent it.

## Electronic Supplementary Material

Below is the link to the electronic supplementary material.

Supplementary Material 1

## Data Availability

The datasets generated and/or analyzed during the current study are not publicly available as analyses are continuing but will be made available to qualified researchers subject to the terms of a data use agreement.

## Abbreviations

aPD: Adjusted prevalence difference
CI: Confidence interval
PP: Percentage point
SD: Standard deviation
