# Supplementary material for: Support for authoritarianism and use of force by and against the federal government in the United States in mid-2025: findings from a nationally representative survey
Source: Inj Epidemiol. 2026 May 26;13:41. doi: 10.1186/s40621-026-00689-y (PMC13214292; doi:10.1186/s40621-026-00689-y)
Supplement: Supplementary file 1 — Supplementary Material 1 [file 40621_2026_689_MOESM1_ESM.pdf]

Supplement

Support for Authoritarianism and Use of Force by and Against the Federal Government in the United States in Mid-2025: Findings from a Nationally Representative Survey

Garen J. Wintemute, MD, MPH; Andrew Crawford, PhD; Elizabeth A. Tomsich, PhD; Mona A. Wright, MPH; Aaron B. Shev, PhD; Daniel J. Tancredi, PhD; Julia P. Schleimer, PhD, MPH; Veronica A. Pear, PhD, MPH, MA; Sonia L. Robinson, PhD, MPH

This supplement has been provided by the authors to give readers additional information about the work.

Page	Title
2	Questions that supplied data for this study
6	Additional methods text
7	Additional results text
8	Figure S1. Flowchart of survey assignment and completion counts for Waves 1-4 in 2022-2025
9	Table S1. Sociodemographic characteristics of respondents
11	Table S2. Sociodemographic characteristics of respondents and non-respondents (unweighted)
13	Table S3. Findings from 2022-2024 on democracy and authoritarianism
14	Table S4. Sociodemographic characteristics and views on democracy and authoritarianism
20	Table S5. Sociodemographic characteristics and support for violence initiated by the federal government
25	Table S6. Sociodemographic characteristics and personal willingness to engage in violence in support of or opposition to the federal government
33	Table S7. Party/MAGA affiliation and views on democracy and authoritarianism
36	Table S8. Party/MAGA affiliation and support for violence initiated by the federal government
39	Table S9. Party/MAGA affiliation and personal willingness to engage in violence in support of or opposition to the federal government
44	References

QUESTIONS THAT SUPPLIED DATA FOR THIS STUDY
Response options are presented here in order from negative to positive (e.g., “not important” to “extremely important”). Respondents were randomized 1:1 to receive responses in that order or the reverse.
In the list below, questions or items that were repeated or adapted from prior surveys contain citations to those surveys.

Democracy and authoritarianism

We’d like to ask you a few questions about the United States as you see it now, in 2025.

Q: How important do you think it is for the United States to remain a democracy?[1]

1. Not important
2. Somewhat important
3. Very important
4. Extremely important

Q: When thinking about democracy in the United States these days, do you believe…?[2]

1. There is a serious threat to our democracy.
2. There may be a threat to our democracy, but it is not serious.
3. There is no threat to our democracy.

Q: How much do you agree or disagree with the following statements about democracy in the United States?

a. Democracy is the best form of government.[3]
c. Having a strong leader for America is more important than having a democracy.
e. We should suspend Congress for a few years so a strong leader can clean up the mess made by politicians in Washington.[4]

1. Do not agree
2. Somewhat agree
3. Strongly agree
4. Very strongly agree

The next items are about your views on goals people might have for the United States. Each item presents two such goals. Please tell us which of the two goals is more important to you.

Q: Which is more important to you…?[5]

1a. Having election outcomes determined democratically
OR
1b. Having political leaders I can trust to look out for my values and interests

Support for violence initiated by the federal government

Now we have a few questions about actions the federal government might take this year.

Q. How much do you agree or disagree with the following statements?

a. The government should use the military to help enforce its policies in the United States.
b. The government should use private armed militia groups to help enforce its policies in the United States.

1. Do not agree
2. Somewhat agree
3. Strongly agree
4. Very strongly agree

Q. And how much do you agree or disagree with these statements? The government should arrest…

a. People who speak out against its policies or the President
b. People who join demonstrations against its policies or the President
c. Reporters and journalists whose stories are critical of its policies or the President

1. Do not agree
2. Somewhat agree
3. Strongly agree
4. Very strongly agree

Personal willingness to engage in violence

Now we have a few questions about the use of force or violence. "Force or violence" means physical force strong enough that it could cause pain or injury to a person. A reminder: your responses will be kept confidential and anonymous.

… [Questions not included in this analysis]

The next questions are about your personal willingness to use force or violence.

… [Questions not included in this analysis]
Q. [I]n a situation where you think force or violence is justified to advance an important political objective, how willing would you personally be to use force or violence against a person because they are…

a. A person who speaks out against government policies or the President
b. A person who joins demonstrations against government policies or the President
c. A reporter or journalist whose stories are critical of government policies or the President

1. Not willing
2. Somewhat willing
3. Very willing
4. Completely willing

Q. This question turns things around: in a situation where you think force or violence is justified to advance an important political objective, how willing would you personally be to use force or violence against a person because they are…

a. A person who speaks out in favor of government policies or the President
b. A person who joins demonstrations in favor of government policies or the President
c. A reporter or journalist whose stories support government policies or the President

1. Not willing
2. Somewhat willing
3. Very willing
4. Completely willing

Q. In general, in a situation where you think force or violence is justified to advance an important political objective, how willing would you personally be to use force or violence this year…

a. To support the government's enforcement of its policies
b. To oppose the government's enforcement of its policies

1. Not willing
2. Somewhat willing
3. Very willing
4. Completely willing

Political party and MAGA movement affiliation

Q: Generally speaking, do you think of yourself as...Select one answer only.

1. Republican
2. Democrat
3. Independent
6. Something else

(Asked if Republican)
Q: Would you call yourself a...Select one answer only.

1. Strong Republican
2. Not very strong Republican

(Asked if Democrat)
Q: Would you call yourself a...Select one answer only.

1. Strong Democrat
2. Not very strong Democrat

(Asked if Independent or Something else)
Q: Do you think of yourself as closer to the...Select one answer only.

1. Republican Party
2. Democratic Party
3. Do not lean either way

(Asked if Republican OR Leans Republican)
Q: Do you think of yourself as a MAGA Republican?

1. No
2. Yes

(Asked if Not MAGA Republican OR Democrat/Lean Democrat OR Do not lean either way)
Q: Do you think of yourself as a supporter of the MAGA movement?

1. No
2. Yes

ADDITIONAL METHODS TEXT

Variables for the linear regression model used to compute aPDs were specified as follows: age (continuous), gender (male, female, other), race and ethnicity (non-Hispanic white, non-Hispanic Black, Hispanic, Asian American or Pacific Islander, and other), education (less than high school, high school, some college/associate degree, bachelor's degree, master's degree or higher), income (<10,000, 10,000-24,999, 25,000-49,999, 50,000-74,999, 75,000-99,999, 100,000-149,999, ≥150,000), Census division (New England, Mid-Atlantic, East-North Central, West-North Central, South Atlantic, East-South Central, West-South Central, Mountain, Pacific), marital status (now married, widowed, divorced or separated, never married), home ownership (yes vs no), living in an urban census tract (yes vs no), firearm ownership (firearm owner, living in a household with a firearm owner, non-owner), total drinks per week (continuous), military service (yes vs no), and history of a non-traffic arrest (yes vs no).

ADDITIONAL RESULTS TEXT

For 1 outcome item, nonresponses exceeded 3%:

Q: Which is more important to you…?

1a. Having election outcomes determined democratically
OR
1b. Having political leaders I can trust to look out for my values and interests

Nonresponse = 3.6%.

Figure S1. Flowchart of survey assignment and completion counts for Waves 1-4 in 2022-2025

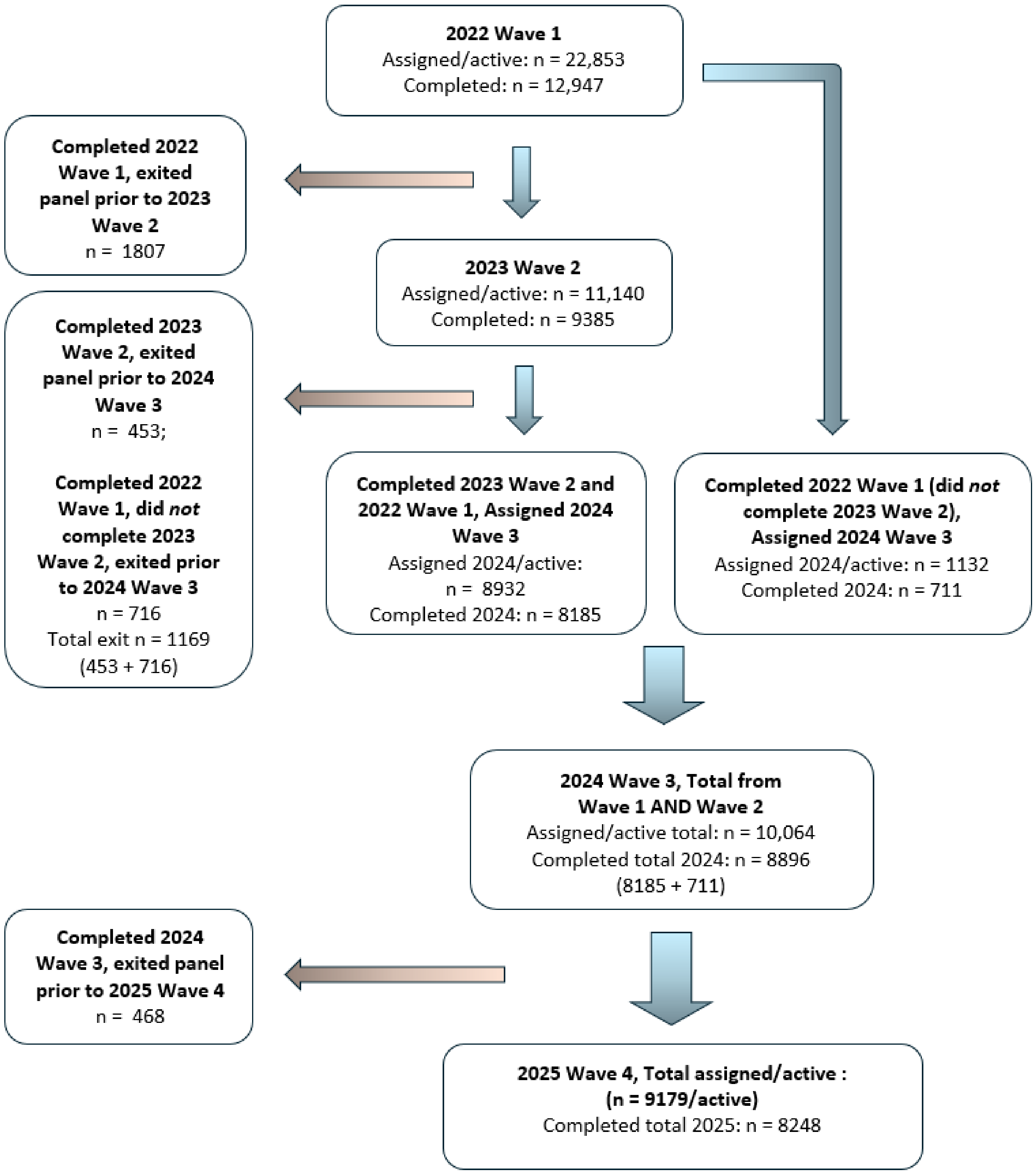

Table S1. Sociodemographic characteristics of respondents

Characteristic	Prevalence 2025 (n=8248)		Estimated N of adults in US*
	Unweighted n	Weighted % (95% CI)	N (95% CI) (in millions)
Age			
18-24	138	5.6 (4.7, 6.7)	15.0 (12.6, 17.8)
25-34	631	16.7 (15.5, 18.1)	44.7 (41.3, 48.4)
35-44	977	16.6 (15.5, 17.7)	44.2 (41.3, 47.3)
45-54	1077	15.3 (14.3, 16.3)	40.8 (38.1, 43.6)
55-64	1672	18.5 (17.5, 19.6)	49.4 (46.7, 52.3)
65-74	2089	15.5 (14.7, 16.3)	41.3 (39.2, 43.5)
75+	1664	11.8 (11.1, 12.6)	31.5 (29.6, 33.6)
Non-response	0	0.0 (0.0, 0.0)	0.0 (0.0, 0.0)
Gender			
Male	4697	47.1 (45.6, 48.5)	125.7 (121.8, 129.6)
Female	3390	50.7 (49.2, 52.2)	135.3 (131.4, 139.2)
Transgender	42	0.5 (0.3, 0.7)	1.2 (0.8, 1.8)
Non-binary	55	0.8 (0.6, 1.1)	2.2 (1.6, 3.0)
Other	17	0.3 (0.1, 0.5)	0.7 (0.4, 1.3)
Non-response	47	0.7 (0.5, 1.1)	1.9 (1.2, 2.9)
Race/Ethnicity			
White, Non-Hispanic	6154	62.7 (61.2, 64.3)	167.5 (163.4, 171.5)
Black, Non-Hispanic	683	12.0 (11.0, 13.1)	32.0 (29.3, 35.0)
Hispanic, any race	870	16.9 (15.7, 18.2)	45.1 (41.8, 48.6)
American Indian or Alaska Native, Non-Hispanic	44	1.1 (0.8, 1.7)	3.0 (2.0, 4.5)
Asian American or Pacific Islander, non-Hispanic	241	5.4 (4.6, 6.2)	14.3 (12.3, 16.6)
Some other race, Non-Hispanic	16	0.1 (0.1, 0.2)	0.3 (0.2, 0.6)
2+ Races, Non-Hispanic	240	1.8 (1.4, 2.2)	4.8 (3.8, 5.9)
Non-response	0	0.0 (0.0, 0.0)	0.0 (0.0, 0.0)
Marital status			
Now married	5245	57.1 (55.6, 58.6)	152.5 (148.5, 156.5)
Widowed	604	5.0 (4.5, 5.6)	13.3 (12.0, 14.8)
Divorced	897	8.5 (7.8, 9.2)	22.6 (20.8, 24.5)
Separated	120	2.1 (1.6, 2.6)	5.6 (4.4, 7.0)
Never married	1382	27.3 (25.9, 28.8)	72.9 (69.1, 77.0)
Non-response	0	0.0 (0.0, 0.0)	0.0 (0.0, 0.0)

Table S1, continued.

Characteristic	Prevalence 2025 (n=8248)		Estimated N of adults in US*
	Unweighted n	Weighted % (95% CI)	N (95% CI) (in millions)
Education			
No high school diploma or GED	287	6.9 (6.0, 7.9)	18.4 (16.1, 21.0)
High school graduate (diploma, GED)	1669	27.2 (25.8, 28.6)	72.5 (68.9, 76.3)
Some college or Associate's degree	2471	28.3 (27.0, 29.6)	75.6 (72.2, 79.1)
Bachelor's degree	2105	21.1 (20.0, 22.2)	56.3 (53.3, 59.3)
Master’s degree or higher	1716	16.6 (15.6, 17.6)	44.2 (41.7, 46.9)
Non-response	0	0.0 (0.0, 0.0)	0.0 (0.0, 0.0)
Household Income			
Less than $10,000	196	4.2 (3.6, 5.0)	11.3 (9.5, 13.5)
$10,000 to $24,999	570	8.2 (7.4, 9.1)	21.8 (19.6, 24.2)
$25,000 to $49,999	1253	16.4 (15.3, 17.6)	43.9 (40.9, 47.0)
$50,000 to $74,999	1262	13.9 (12.9, 14.9)	37.1 (34.5, 39.8)
$75,000 to $99,999	1246	13.2 (12.3, 14.2)	35.3 (32.9, 38.0)
$100,000 to $149,999	1674	18.9 (17.8, 20.0)	50.4 (47.5, 53.4)
$150,000 or more	2047	25.2 (23.9, 26.4)	67.2 (63.9, 70.5)
Non-response	0	0.0 (0.0, 0.0)	0.0 (0.0, 0.0)
Employment			
Working - as a paid employee	3902	53.6 (52.1, 55.0)	143.0 (139.1, 146.9)
Working - self-employed	606	7.1 (6.4, 7.9)	19.1 (17.1, 21.2)
Not working - on temporary layoff from a job	32	0.5 (0.3, 0.8)	1.4 (0.9, 2.2)
Not working - looking for work	228	5.0 (4.2, 5.8)	13.3 (11.3, 15.6)
Not working - retired	2877	21.3 (20.3, 22.3)	56.7 (54.2, 59.4)
Not working - disabled	237	4.4 (3.8, 5.1)	11.8 (10.1, 13.7)
Not working - other	366	8.1 (7.2, 9.2)	21.8 (19.3, 24.4)
Non-response	0	0.0 (0.0, 0.0)	0.0 (0.0, 0.0)
Census division**			
New England	340	4.7 (4.1, 5.3)	12.5 (10.9, 14.3)
Mid-Atlantic	900	12.4 (11.5, 13.5)	33.2 (30.7, 35.9)
East-North Central	1228	14.5 (13.5, 15.5)	38.6 (36.0, 41.4)
West-North Central	597	6.4 (5.8, 7.2)	17.2 (15.5, 19.1)
South Atlantic	1624	20.4 (19.3, 21.7)	54.6 (51.4, 57.9)
East-South Central	463	5.9 (5.2, 6.7)	15.8 (14.0, 17.8)
West-South Central	854	11.8 (10.8, 12.8)	31.5 (28.9, 34.3)
Mountain	733	7.7 (6.9, 8.5)	20.5 (18.5, 22.7)
Pacific	1509	16.1 (15.1, 17.2)	43.1 (40.4, 45.9)
Non-response	0	0.0 (0.0, 0.0)	0.0 (0.0, 0.0)

* Sample population extrapolations are based on the estimated US adult (≥age 18) population, *N* = 266,978,268 as of July 1, 2024 [6].

† Census division values were updated for 2025; other values are from 2022.

Table S2. Sociodemographic characteristics of respondents and non-respondents (unweighted)*

Characteristics	Respondents (n=8248)		Non-respondents (n = 931)	
	Unweighted n	Unweighted %	Unweighted n	Unweighted %
Age				
18-24	138	1.7	56	6.0
25-34	631	7.7	115	12.4
35-44	977	11.8	168	18.0
45-54	1077	13.1	136	14.6
55-64	1672	20.3	174	18.7
65-74	2089	25.3	177	19.0
75+	1664	20.2	105	11.3
Non-response	0	0.0	0	0.0
Gender				
Male	4780	58.9	457	49.1
Female	3468	42.0	474	50.9
Non-response	0	0.0	0	0.0
Race and ethnicity				
Black, non-Hispanic	670	8.1	88	9.5
Hispanic, any race	889	10.8	157	16.9
White, non-Hispanic	6149	74.6	619	66.5
Other, non-Hispanic	301	3.6	44	4.7
2+races, non-Hispanic	239	2.9	23	2.5
Non-response	0	0.0	0	0.0
Marital status				
Now married	5245	63.6	569	61.1
Widowed	604	7.3	48	5.2
Divorced	897	10.9	98	10.5
Separated	120	1.5	20	2.1
Never married	1382	16.8	196	21.1
Non-response	0	0.0	0	0.0
Education				
No high school diploma or GED	287	3.5	59	6.3
High school graduate (diploma, GED)	1669	20.2	220	23.6
Some college or Associate's degree	2471	30	280	30.1
Bachelor's degree	2105	25.5	219	23.5
Master’s degree or higher	1716	20.8	153	16.4
Non-response	0	0.0	0	0.0

Table S2, continued.

Characteristics	Respondents (n=8248)		Non-respondents (n = 931)	
	Unweighted n	Unweighted %	Unweighted n	Unweighted %
Household Income				
Less than $10,000	196	2.4	37	4.0
$10,000 to $24,999	570	6.9	98	10.5
$25,000 to $49,999	1253	15.2	173	18.6
$50,000 to $74,999	1262	15.3	139	14.9
$75,000 to $99,999	1246	15.1	136	14.6
$100,000 to $149,999	1674	20.3	180	19.3
$150,000 or more	2047	24.8	168	18.0
Non-response	0	0.0	0	0.0
Employment				
Working full-time	3268	39.6	408	43.8
Working part-time	973	11.8	142	15.3
Not working	4007	48.6	381	40.9
Non-response	0	0.0	0	0.0
Census division				
New England	340	4.1	30	3.2
Mid-Atlantic	900	10.9	101	10.8
East-North Central	1228	14.9	144	15.5
West-North Central	597	7.2	55	5.9
South Atlantic	1624	19.7	190	20.4
East-South Central	463	5.6	56	6.0
West-South Central	854	10.4	96	10.3
Mountain	733	8.9	71	7.6
Pacific	1509	18.3	188	20.2
Non-response	0	0.0	0	0.0

* Most values are as of 2022; Census division values were updated for 2025. Genders other than male and female were not available for nonrespondents. Mean (SD) ages were as follows: respondents, 48.5 (24.9) years; non-respondents, 52.6 (17.6) years.

Table S3. Findings from 2022-2024 on democracy and authoritarianism*

The United States as you see it now	2022 Respondents (n = 12,947)		2023 Respondents (n = 9385)		2024 Respondents (n = 8896)	
	Unweighted n	Weighted % (95% CI)	Unweighted n	Weighted % (95% CI)	Unweighted n	Weighted % (95% CI)
How important do you think it is for the United States to remain a democracy?						
Not important	191	2.1 (1.8, 2.5)	261	4.0 (3.4, 4.6)	193	3.5 (2.9, 4.2)
Somewhat important	659	7.7 (7.0, 8.4)	570	9.7 (8.8, 10.7)	411	7.6 (6.8, 8.5)
Very or extremely important	12003	89.0 (88.2, 89.8)	8448	84.6 (83.4, 85.7)	8208	87.5 (86.3, 88.5)
Non-response	94	1.2 (0.9, 1.4)	106	1.7 (1.3, 2.2)	84	1.4 (1.1, 1.9)
When thinking about democracy in the United States these days, do you believe…						
There is a serious threat to our democracy.	9409	67.4 (66.3, 68.5)	6452	62.3 (60.9, 63.7)	6256	64.8 (63.4, 66.2)
There may be a threat to our democracy, but it is not serious.	2640	23.5 (22.5, 24.5)	2253	28.0 (26.8, 29.3)	1953	25.9 (24.6, 27.2)
There is no threat to our democracy.	780	7.7 (7.0, 8.4)	529	7.0 (6.2, 7.8)	573	7.3 (6.5, 8.1)
Non-response	118	1.4 (1.1, 1.7)	151	2.6 (2.1, 3.1)	114	2.0 (1.6, 2.5)
How much do you agree or disagree with the following statements about democracy in the United States?						
Democracy is the best form of government.						
Do not agree	595	5.8 (5.2, 6.4)	531	7.5 (6.7, 8.4)	493	7.2 (6.4, 8.0)
Somewhat agree	2396	23.1 (22.1, 24.1)	1765	24.1 (22.8, 25.3)	1507	22.0 (20.7, 23.3)
Strongly or very strongly agree	9823	69.5 (68.5, 70.6)	6948	65.9 (64.5, 67.3)	6775	68.7 (67.3, 70.1)
Non-response	133	1.6 (1.3, 1.9)	141	2.5 (2.0, 3.0)	121	2.2 (1.7, 2.7)
Having a strong leader for America is more important than having a democracy.						
Do not agree	7921	56.2 (55.1, 57.3)	6219	59.6 (58.2, 61.0)	6076	63.0 (61.6, 64.4)
Somewhat agree	2628	23.0 (22.1, 24.0)	1685	21.7 (20.5, 22.9)	1403	18.8 (17.6, 20.0)
Strongly or very strongly agree	2254	19.1 (18.2, 20.0)	1333	16.1 (15.0, 17.1)	1280	15.9 (14.9, 17.1)
Non-response	144	1.6 (1.3, 2.0)	148	2.6 (2.1, 3.2)	137	2.3 (1.8, 2.8)

* These data were initially published as part of Table 2 in Wintemute GJ, Crawford A, Tomsich EA, Pear VA. Trends in views of democracy and society and support for political violence in the USA, 2022-2024: findings from a nationally representative survey. Inj Epidemiol. 2025;12(1):4 [8]. Questions were asked of all participants.

Table S4. Sociodemographic characteristics and views on democracy and authoritarianism*

Characteristics	How important do you think it is for the United States to remain a democracy?					
	Not important		Somewhat important		Very or extremely important	
	Unweighted n	Weighted % (95% CI)	Unweighted n	Weighted % (95% CI)	Unweighted n	Weighted % (95% CI)
Age						
18-24	8	5.9 (5.2, 6.6)	18	14.6 (11.9, 17.9)	105	79.5 (76.0, 82.6)
25-34	22	4.8 (3.8, 6.0)	52	10.8 (9.8, 11.9)	535	84.0 (82.3, 85.5)
35-44	33	4.1 (3.4, 4.9)	80	10.3 (9.5, 11.1)	830	85.6 (84.4, 86.7)
45-54	26	3.2 (2.8, 3.5)	62	7.4 (7.1, 7.8)	958	89.0 (88.4, 89.6)
55-64	40	2.5 (2.2, 2.8)	57	4.7 (4.3, 5.1)	1517	91.4 (90.8, 91.9)
65-74	34	1.9 (1.8, 2.0)	60	3.8 (3.5, 4.1)	1917	93.3 (92.9, 93.6)
75+	14	1.1 (1.0, 1.1)	20	1.4 (1.1, 1.6)	1574	96.9 (96.6, 97.2)
Gender						
Male	100	3.2 (2.8, 3.6)	148	5.8 (5.5, 6.1)	4330	90.5 (90.0, 91.0)
Female	70	3.1 (2.8, 3.4)	190	8.3 (7.9, 8.7)	2972	87.8 (87.3, 88.3)
Transgender	1	1.4 (1.2, 1.8)	3	11.9 (9.5, 14.8)	36	86.7 (83.5, 89.3)
Non-binary	2	4.1 (3.1, 5.4)	4	5.7 (4.3, 7.5)	49	90.1 (87.1, 92.5)
Other	0	0.0 (0.0, 0.0)	1	3.1 (1.7, 5.4)	16	96.9 (94.6, 98.3)
Race and Ethnicity						
White, Non-Hispanic	112	2.5 (2.3, 2.7)	213	5.7 (5.6, 5.9)	5649	91.1 (90.8, 91.4)
Black, Non-Hispanic	14	3.2 (2.6, 4.0)	42	8.8 (7.9, 9.7)	580	86.7 (85.4, 87.9)
Hispanic, any race	36	5.2 (4.3, 6.3)	65	10.6 (9.7, 11.5)	734	83.7 (82.3, 85.1)
American Indian or Alaska Native, Non-Hispanic	3	7.8 (4.9, 12.2)	4	18.6 (14.9, 22.8)	35	73.7 (67.1, 79.3)
Asian American or Pacific Islander, non-Hispanic	6	3.2 (2.8, 3.6)	10	5.4 (3.1, 9.2)	216	91.3 (87.9, 93.9)
Some other race, Non-Hispanic	0	0.0 (0.0, 0.0)	0	0.0 (0.0, 0.0)	14	88.4 (82.4, 92.5)
2+ Races, Non-Hispanic	6	4.1 (3.5, 4.9)	15	7.9 (6.6, 9.4)	208	87.2 (84.8, 89.3)

Table S4, continued.

Characteristics	When thinking about democracy in the United States these days, do you believe…					
	There is a serious threat to our democracy.		There may be a threat to our democracy, but it is not serious.		There is no threat to our democracy.	
	Unweighted n	Weighted % (95% CI)	Unweighted n	Weighted % (95% CI)	Unweighted n	Weighted % (95% CI)
Age						
18-24	67	51.5 (45.7, 57.2)	47	37.8 (32.8, 43.0)	16	9.9 (8.1, 12.1)
25-34	364	57.0 (54.0, 60.0)	182	31.1 (28.4, 33.9)	61	10.9 (9.5, 12.5)
35-44	568	59.2 (56.8, 61.5)	268	28.8 (26.7, 31.0)	97	10.2 (9.3, 11.1)
45-54	628	60.1 (57.9, 62.4)	280	26.3 (24.4, 28.2)	130	11.6 (10.4, 12.9)
55-64	1003	62.3 (60.4, 64.2)	380	23.7 (22.1, 25.3)	235	13.0 (11.8, 14.2)
65-74	1401	70.8 (69.3, 72.1)	383	18.2 (17.1, 19.4)	230	10.1 (9.4, 11.0)
75+	1106	69.3 (67.4, 71.2)	290	17.4 (15.9, 19.0)	211	12.2 (11.0, 13.5)
Gender						
Male	2811	59.9 (58.8, 61.0)	1112	26.2 (25.2, 27.2)	652	13.0 (12.4, 13.6)
Female	2221	63.9 (62.7, 65.1)	688	24.8 (23.7, 25.8)	310	9.7 (9.1, 10.2)
Transgender	28	52.3 (41.7, 62.7)	7	29.2 (22.8, 36.4)	5	18.5 (11.7, 28.1)
Non-binary	42	79.4 (68.5, 87.3)	10	10.8 (5.5, 20.3)	3	9.7 (4.9, 18.4)
Other	12	86.5 (76.8, 92.5)	2	6.2 (3.4, 10.9)	3	7.4 (4.1, 12.8)
Race and Ethnicity						
White, Non-Hispanic	3792	62.4 (61.5, 63.2)	1397	25.2 (24.4, 25.9)	776	11.3 (10.9, 11.8)
Black, Non-Hispanic	499	70.8 (68.5, 73.0)	103	20.3 (18.3, 22.5)	31	6.5 (6.0, 7.0)
Hispanic, any race	513	57.0 (54.4, 59.6)	203	27.3 (25.1, 29.7)	114	14.4 (13.3, 15.5)
American Indian or Alaska Native, Non-Hispanic	19	43.6 (33.5, 54.3)	14	44.0 (33.8, 54.7)	9	12.4 (6.8, 21.5)
Asian American or Pacific Islander, non-Hispanic	141	57.4 (51.6, 63.0)	63	30.0 (25.1, 35.4)	26	11.7 (8.9, 15.3)
Some other race, Non-Hispanic	10	46.1 (26.2, 67.4)	3	39.6 (21.6, 61.0)	2	14.3 (9.2, 21.5)
2+ Races, Non-Hispanic	163	71.7 (65.8, 77.0)	47	19.2 (15.0, 24.1)	22	8.9 (7.2, 10.9)

Table S4, continued.

Characteristics	Democracy is the best form of government.					
	Do not agree		Somewhat agree		Strongly or very strongly agree	
	Unweighted n	Weighted % (95% CI)	Unweighted n	Weighted % (95% CI)	Unweighted n	Weighted % (95% CI)
Age						
18-24	18	12.3 (10.4, 14.4)	47	39.2 (33.9, 44.9)	64	46.3 (40.4, 52.3)
25-34	51	10.0 (8.4, 11.7)	178	30.5 (28.0, 33.2)	373	58.0 (55.1, 61.0)
35-44	63	7.0 (6.0, 8.0)	246	27.6 (25.7, 29.7)	630	64.6 (62.4, 66.8)
45-54	62	6.7 (6.0, 7.6)	226	22.7 (21.1, 24.5)	753	69.0 (67.1, 71.0)
55-64	97	5.9 (5.4, 6.4)	254	16.9 (15.7, 18.3)	1273	76.4 (75.0, 77.8)
65-74	105	5.7 (5.4, 6.1)	257	14.8 (13.9, 15.7)	1651	78.5 (77.5, 79.6)
75+	55	3.5 (3.0, 4.0)	154	9.7 (8.7, 10.9)	1404	86.3 (85.1, 87.4)
Gender						
Male	262	6.7 (6.2, 7.2)	666	19.1 (18.2, 20.0)	3654	73.4 (72.4, 74.4)
Female	173	7.1 (6.5, 7.6)	654	24.0 (23.0, 25.0)	2400	67.6 (66.5, 68.7)
Transgender	3	4.7 (1.5, 14.0)	11	30.9 (22.9, 40.3)	26	64.4 (54.3, 73.3)
Non-binary	5	10.7 (8.1, 14.0)	17	39.9 (26.9, 54.6)	32	48.1 (34.9, 61.6)
Other	1	1.9 (1.1, 3.4)	5	16.8 (8.2, 31.4)	11	81.3 (66.0, 90.7)
Race and Ethnicity						
White, Non-Hispanic	315	5.8 (5.5, 6.1)	898	19.1 (18.4, 19.8)	4764	74.1 (73.4, 74.9)
Black, Non-Hispanic	38	7.1 (6.2, 8.1)	156	30.4 (28.0, 32.8)	438	60.2 (57.6, 62.8)
Hispanic, any race	62	10.1 (9.0, 11.4)	194	25.9 (23.9, 28.0)	574	63.0 (60.5, 65.4)
American Indian or Alaska Native, Non-Hispanic	4	16.5 (12.5, 21.6)	16	39.4 (31.2, 48.3)	22	44.1 (32.8, 56.0)
Asian American or Pacific Islander, non-Hispanic	12	6.8 (4.5, 10.2)	49	19.7 (15.9, 24.2)	172	73.5 (68.4, 78.0)
Some other race, Non-Hispanic	1	9.6 (6.2, 14.6)	3	16.2 (9.6, 26.1)	11	74.2 (61.0, 84.1)
2+ Races, Non-Hispanic	19	9.4 (7.8, 11.2)	46	25.0 (17.8, 33.9)	167	65.4 (57.0, 73.0)

Table S4, continued.

Characteristics	Having a strong leader for America is more important than having a democracy.					
	Do not agree		Somewhat agree		Strongly or very strongly agree	
	Unweighted n	Weighted % (95% CI)	Unweighted n	Weighted % (95% CI)	Unweighted n	Weighted % (95% CI)
Age						
18-24	77	56.7 (51.5, 61.7)	27	23.0 (19.9, 26.4)	25	18.1 (16.1, 20.4)
25-34	417	65.1 (62.6, 67.6)	114	20.3 (18.3, 22.3)	71	13.3 (12.3, 14.4)
35-44	644	65.5 (63.5, 67.5)	167	18.4 (17.1, 19.7)	127	15.2 (14.0, 16.4)
45-54	696	64.2 (62.2, 66.2)	184	18.2 (16.8, 19.8)	159	15.9 (15.0, 17.0)
55-64	1135	67.3 (65.6, 69.0)	276	17.7 (16.4, 19.1)	210	14.0 (13.2, 14.9)
65-74	1458	68.7 (67.4, 70.0)	271	14.6 (13.7, 15.6)	285	15.7 (14.9, 16.5)
75+	1183	69.7 (67.8, 71.4)	201	13.1 (12.1, 14.3)	216	15.8 (14.4, 17.3)
Gender						
Male	3335	69.3 (68.4, 70.2)	654	16.0 (15.3, 16.6)	579	13.5 (13.0, 14.0)
Female	2154	62.9 (61.8, 64.0)	567	19.4 (18.5, 20.2)	502	16.4 (15.8, 17.0)
Transgender	35	75.1 (69.2, 80.1)	2	4.6 (3.7, 5.8)	3	20.3 (16.2, 25.1)
Non-binary	43	76.8 (67.5, 84.0)	9	14.3 (8.5, 23.2)	2	7.7 (5.8, 10.0)
Other	15	89.9 (82.6, 94.4)	2	10.1 (5.6, 17.4)	0	0.0 (0.0, 0.0)
Race and Ethnicity						
White, Non-Hispanic	4323	69.0 (68.3, 69.7)	876	16.3 (15.7, 16.8)	758	13.5 (13.1, 13.9)
Black, Non-Hispanic	401	55.5 (52.8, 58.2)	120	22.0 (20.1, 24.1)	114	20.4 (18.9, 22.0)
Hispanic, any race	548	64.4 (62.2, 66.5)	148	18.2 (16.7, 19.8)	134	16.4 (15.3, 17.5)
American Indian or Alaska Native, Non-Hispanic	18	53.7 (43.6, 63.5)	13	20.5 (14.0, 28.9)	11	25.9 (20.7, 31.7)
Asian American or Pacific Islander, non-Hispanic	147	62.2 (57.0, 67.2)	47	20.7 (16.7, 25.4)	38	16.8 (14.3, 19.6)
Some other race, Non-Hispanic	13	81.9 (49.9, 95.4)	1	13.5 (2.1, 53.4)	1	4.7 (3.0, 7.1)
2+ Races, Non-Hispanic	160	67.3 (61.1, 73.0)	35	18.7 (15.2, 22.9)	37	13.7 (11.0, 17.0)

Table S4, continued.

Characteristics	We should suspend Congress for a few years so a strong leader can clean up the mess made by politicians in Washington.					
	Do not agree		Somewhat agree		Strongly or very strongly agree	
	Unweighted n	Weighted % (95% CI)	Unweighted n	Weighted % (95% CI)	Unweighted n	Weighted % (95% CI)
Age						
18-24	85	62.8 (58.3, 67.1)	27	22.2 (19.4, 25.3)	16	12.2 (10.9, 13.7)
25-34	459	70.1 (67.9, 72.2)	90	17.0 (15.5, 18.6)	53	11.2 (10.2, 12.3)
35-44	681	67.7 (65.8, 69.6)	146	18.0 (16.6, 19.6)	108	12.6 (11.8, 13.5)
45-54	754	69.5 (67.7, 71.2)	155	16.2 (15.0, 17.5)	129	12.8 (11.9, 13.8)
55-64	1243	72.3 (70.8, 73.7)	228	16.1 (15.0, 17.2)	151	10.4 (9.7, 11.2)
65-74	1544	72.1 (70.9, 73.4)	249	13.9 (13.0, 14.9)	224	13.1 (12.4, 13.9)
75+	1312	77.2 (75.6, 78.8)	167	11.6 (10.5, 12.8)	124	9.6 (8.6, 10.8)
Gender						
Male	3691	75.1 (74.3, 75.8)	499	13.5 (13.0, 14.0)	384	10.3 (9.8, 10.7)
Female	2265	66.9 (65.9, 67.9)	546	18.3 (17.5, 19.0)	409	13.1 (12.6, 13.6)
Transgender	35	75.1 (69.2, 80.1)	2	18.5 (14.8, 22.8)	3	6.5 (5.2, 8.1)
Non-binary	47	84.1 (78.4, 88.5)	4	7.3 (4.5, 11.6)	3	7.4 (5.6, 9.7)
Other	14	84.2 (73.1, 91.3)	2	11.4 (6.3, 19.6)	1	4.4 (2.5, 7.7)
Race and Ethnicity						
White, Non-Hispanic	4715	75.1 (74.6, 75.7)	708	13.1 (12.7, 13.6)	546	10.5 (10.2, 10.8)
Black, Non-Hispanic	415	59.7 (57.1, 62.2)	112	19.5 (18.0, 21.2)	106	18.3 (16.7, 20.0)
Hispanic, any race	565	63.0 (60.7, 65.2)	165	23.3 (21.7, 25.1)	93	11.4 (10.4, 12.4)
American Indian or Alaska Native, Non-Hispanic	27	61.2 (52.6, 69.2)	8	22.3 (17.5, 28.1)	7	16.4 (13.2, 20.2)
Asian American or Pacific Islander, non-Hispanic	170	69.7 (65.2, 73.8)	36	18.2 (14.8, 22.1)	27	12.1 (10.5, 14.0)
Some other race, Non-Hispanic	12	86.2 (67.6, 94.9)	0	0.0 (0.0, 0.0)	3	13.8 (5.1, 32.4)
2+ Races, Non-Hispanic	174	70.9 (65.0, 76.1)	33	17.5 (14.0, 21.7)	23	10.4 (8.4, 12.7)

Table S4, continued.

Characteristics	Which is more important to you…?			
	Having election outcomes determined democratically		Having political leaders I can trust to look out for my values and interests	
	Unweighted n	Weighted % (95% CI)	Unweighted n	Weighted % (95% CI)
Age				
18-24	73	51.4 (45.6, 57.1)	51	41.4 (36.1, 47.0)
25-34	384	58.2 (55.3, 61.1)	201	35.0 (32.3, 37.7)
35-44	587	58.2 (55.8, 60.5)	313	35.0 (32.9, 37.3)
45-54	699	61.9 (59.6, 64.2)	321	34.2 (32.0, 36.5)
55-64	1101	64.7 (62.8, 66.5)	486	31.1 (29.4, 32.9)
65-74	1377	63.6 (62.0, 65.2)	596	33.2 (31.7, 34.7)
75+	1155	67.6 (65.5, 69.5)	437	29.9 (27.9, 31.9)
Gender				
Male	3275	66.8 (65.7, 67.8)	1208	28.6 (27.6, 29.6)
Female	1987	56.3 (55.1, 57.6)	1162	38.7 (37.5, 39.9)
Transgender	32	67.0 (58.5, 74.6)	7	18.1 (12.5, 25.4)
Non-binary	48	84.6 (79.0, 89.0)	6	14.5 (10.3, 19.9)
Other	10	75.0 (57.5, 87.0)	7	25.0 (13.0, 42.5)
Race and Ethnicity				
White, Non-Hispanic	4158	65.8 (65.0, 66.5)	1747	32.1 (31.4, 32.9)
Black, Non-Hispanic	366	48.4 (45.5, 51.4)	225	40.6 (38.0, 43.3)
Hispanic, any race	510	55.6 (52.9, 58.2)	269	33.9 (31.5, 36.3)
American Indian or Alaska Native, Non-Hispanic	18	33.8 (23.3, 46.2)	21	51.4 (40.8, 61.9)
Asian American or Pacific Islander, non-Hispanic	162	64.4 (58.5, 69.9)	64	31.9 (26.5, 37.8)
Some other race, Non-Hispanic	10	55.5 (33.8, 75.3)	5	44.5 (24.7, 66.2)
2+ Races, Non-Hispanic	152	64.4 (57.6, 70.6)	74	32.2 (26.5, 38.5)

* Characteristics are as of participation in Wave 1 in 2022. Questions were asked of all participants.

Table S5. Sociodemographic characteristics and support for violence initiated by the federal government*

Characteristics	The government should use the military to help enforce its policies in the United States.					
	Do not agree		Somewhat agree		Strongly or very strongly agree	
	Unweighted n	Weighted % (95% CI)	Unweighted n	Weighted % (95% CI)	Unweighted n	Weighted % (95% CI)
Age						
18-24	86	62.7 (57.9, 67.2)	30	23.8 (20.5, 27.4)	14	12.6 (10.7, 14.7)
25-34	414	65.6 (62.9, 68.1)	127	22.4 (20.2, 24.9)	63	10.9 (10.2, 11.6)
35-44	635	66.9 (64.8, 68.8)	221	22.6 (20.9, 24.3)	76	8.9 (8.4, 9.6)
45-54	683	66.5 (64.5, 68.5)	246	22.5 (20.8, 24.3)	112	9.8 (9.2, 10.6)
55-64	1102	67.0 (65.3, 68.7)	379	23.4 (21.9, 25.0)	145	9.1 (8.5, 9.7)
65-74	1385	68.3 (66.9, 69.6)	444	21.9 (20.7, 23.1)	191	9.4 (8.8, 10.0)
75+	1093	65.6 (63.6, 67.5)	372	23.9 (22.2, 25.6)	140	9.3 (8.4, 10.3)
Gender						
Male	3206	69.4 (68.4, 70.3)	990	20.7 (19.9, 21.5)	384	9.1 (8.8, 9.4)
Female	2074	63.3 (62.2, 64.4)	803	25.1 (24.1, 26.1)	351	10.5 (10.1, 10.9)
Transgender	32	67.5 (59.7, 74.5)	7	28.2 (22.0, 35.2)	1	4.3 (3.5, 5.3)
Non-binary	45	81.2 (72.1, 87.8)	6	7.9 (3.1, 18.7)	3	9.7 (7.3, 12.7)
Other	13	84.5 (73.5, 91.4)	4	15.5 (8.6, 26.5)	0	0.0 (0.0, 0.0)
Race and Ethnicity						
White, Non-Hispanic	3969	65.2 (64.5, 66.0)	1444	24.3 (23.6, 24.9)	564	9.5 (9.3, 9.8)
Black, Non-Hispanic	474	69.7 (67.6, 71.7)	96	16.4 (15.0, 18.0)	63	12.3 (11.3, 13.4)
Hispanic, any race	597	68.9 (66.9, 70.9)	169	22.2 (20.5, 24.0)	64	8.1 (7.6, 8.6)
American Indian or Alaska Native, Non-Hispanic	23	51.0 (40.6, 61.3)	13	36.6 (28.7, 45.3)	6	12.4 (10.0, 15.3)
Asian American or Pacific Islander, non-Hispanic	170	70.2 (65.1, 74.7)	40	17.9 (13.7, 23.0)	21	11.0 (9.8, 12.4)
Some other race, Non-Hispanic	14	98.0 (97.0, 98.7)	1	2.0 (1.3, 3.0)	0	0.0 (0.0, 0.0)
2+ Races, Non-Hispanic	151	62.2 (53.6, 70.2)	56	25.8 (18.1, 35.2)	23	11.4 (9.3, 13.9)

Table S5, continued.

Characteristics	The government should use private armed militia groups to help enforce its policies in the United States.					
	Do not agree		Somewhat agree		Strongly or very strongly agree	
	Unweighted n	Weighted % (95% CI)	Unweighted n	Weighted % (95% CI)	Unweighted n	Weighted % (95% CI)
Age						
18-24	104	75.9 (72.8, 78.7)	16	14.3 (12.5, 16.4)	10	8.9 (7.9, 9.9)
25-34	500	78.7 (77.3, 80.2)	61	12.2 (11.2, 13.2)	42	7.6 (7.1, 8.1)
35-44	795	82.1 (81.1, 83.1)	83	9.5 (8.9, 10.2)	52	6.6 (6.2, 6.9)
45-54	894	83.8 (82.9, 84.6)	101	10.9 (10.3, 11.6)	46	4.2 (4.0, 4.5)
55-64	1464	88.0 (87.3, 88.7)	132	9.1 (8.5, 9.8)	31	2.2 (2.1, 2.4)
65-74	1853	90.9 (90.4, 91.3)	127	6.7 (6.3, 7.1)	44	2.0 (1.8, 2.1)
75+	1498	91.2 (90.7, 91.7)	75	5.0 (4.6, 5.3)	30	2.5 (2.4, 2.6)
Gender						
Male	4172	87.0 (86.6, 87.4)	268	7.5 (7.2, 7.8)	140	4.6 (4.5, 4.7)
Female	2800	83.0 (82.5, 83.5)	316	11.2 (10.8, 11.6)	112	4.5 (4.4, 4.6)
Transgender	37	92.1 (90.2, 93.7)	3	7.9 (6.3, 9.8)	0	0.0 (0.0, 0.0)
Non-binary	50	90.2 (85.6, 93.4)	3	7.5 (4.7, 11.8)	1	1.1 (0.8, 1.4)
Other	16	95.6 (92.3, 97.5)	1	4.4 (2.5, 7.7)	0	0.0 (0.0, 0.0)
Race and Ethnicity						
White, Non-Hispanic	5396	87.4 (87.1, 87.7)	412	8.1 (7.8, 8.4)	164	3.4 (3.3, 3.4)
Black, Non-Hispanic	543	79.3 (78.0, 80.5)	58	11.6 (10.9, 12.4)	35	7.8 (7.4, 8.3)
Hispanic, any race	709	80.6 (79.3, 81.8)	89	13.8 (12.8, 14.9)	34	4.9 (4.7, 5.2)
American Indian or Alaska Native, Non-Hispanic	33	73.0 (66.8, 78.3)	6	13.7 (11.1, 16.9)	2	8.7 (7.0, 10.7)
Asian American or Pacific Islander, non-Hispanic	205	84.7 (82.8, 86.4)	14	6.2 (5.5, 7.0)	11	7.6 (6.8, 8.6)
Some other race, Non-Hispanic	14	88.4 (82.4, 92.5)	1	11.6 (7.5, 17.6)	0	0.0 (0.0, 0.0)
2+ Races, Non-Hispanic	208	87.2 (84.1, 89.8)	15	5.5 (3.9, 7.6)	9	7.2 (5.6, 9.1)

Table S5, continued.

Characteristics	The government should arrest people who speak out against its policies or the President.					
	Do not agree		Somewhat agree		Strongly or very strongly agree	
	Unweighted n	Weighted % (95% CI)	Unweighted n	Weighted % (95% CI)	Unweighted n	Weighted % (95% CI)
Age						
18-24	103	77.6 (74.7, 80.3)	17	12.4 (10.7, 14.2)	10	9.2 (8.1, 10.3)
25-34	531	84.0 (82.8, 85.1)	48	9.8 (9.1, 10.5)	26	5.1 (4.5, 5.8)
35-44	787	81.4 (80.3, 82.5)	89	10.4 (9.8, 11.1)	58	6.5 (5.9, 7.1)
45-54	900	83.4 (82.4, 84.3)	80	9.0 (8.6, 9.5)	56	5.9 (5.4, 6.5)
55-64	1458	87.1 (86.5, 87.8)	111	8.2 (7.9, 8.6)	58	4.4 (4.0, 4.8)
65-74	1804	86.7 (86.1, 87.4)	129	7.6 (7.3, 7.9)	84	5.0 (4.5, 5.6)
75+	1417	85.1 (84.0, 86.1)	117	8.4 (7.6, 9.3)	71	5.3 (4.9, 5.7)
Gender						
Male	4093	86.2 (85.8, 86.6)	305	8.1 (7.8, 8.4)	181	4.9 (4.7, 5.1)
Female	2776	82.8 (82.2, 83.3)	274	9.8 (9.5, 10.1)	177	6.2 (5.9, 6.6)
Transgender	35	72.9 (63.7, 80.4)	4	25.1 (17.7, 34.2)	1	2.1 (1.7, 2.6)
Non-binary	51	88.6 (83.8, 92.1)	3	10.1 (6.8, 14.7)	0	0.0 (0.0, 0.0)
Other	15	89.9 (82.6, 94.4)	1	4.4 (2.5, 7.7)	0	0.0 (0.0, 0.0)
Race and Ethnicity						
White, Non-Hispanic	5371	88.0 (87.7, 88.3)	396	7.1 (6.9, 7.3)	211	4.1 (3.9, 4.3)
Black, Non-Hispanic	498	70.6 (68.7, 72.5)	69	15.6 (14.5, 16.8)	61	10.6 (9.6, 11.8)
Hispanic, any race	690	80.8 (79.6, 82.0)	81	11.3 (10.5, 12.1)	57	6.8 (6.2, 7.3)
American Indian or Alaska Native, Non-Hispanic	32	71.2 (64.4, 77.3)	8	21.1 (16.4, 26.7)	2	7.6 (6.2, 9.4)
Asian American or Pacific Islander, non-Hispanic	194	82.5 (80.2, 84.5)	22	10.0 (8.8, 11.2)	17	7.6 (6.5, 8.8)
Some other race, Non-Hispanic	14	97.8 (96.7, 98.6)	1	2.2 (1.4, 3.3)	0	0.0 (0.0, 0.0)
2+ Races, Non-Hispanic	201	85.2 (82.4, 87.6)	14	8.3 (7.0, 9.9)	15	5.9 (5.0, 7.1)

Table S5, continued.

Characteristics	The government should arrest people who join demonstrations against its policies or the President.					
	Do not agree		Somewhat agree		Strongly or very strongly agree	
	Unweighted n	Weighted % (95% CI)	Unweighted n	Weighted % (95% CI)	Unweighted n	Weighted % (95% CI)
Age						
18-24	101	75.8 (72.6, 78.8)	21	17.4 (15.1, 20.0)	8	5.9 (5.2, 6.6)
25-34	507	79.3 (77.9, 80.8)	67	13.2 (12.3, 14.2)	31	6.6 (5.9, 7.3)
35-44	762	79.1 (77.9, 80.3)	111	11.3 (10.6, 12.1)	63	8.1 (7.5, 8.8)
45-54	869	80.3 (79.1, 81.4)	111	12.7 (11.9, 13.5)	58	5.5 (4.9, 6.1)
55-64	1386	82.9 (82.1, 83.7)	167	12.2 (11.6, 12.8)	70	4.5 (4.0, 4.9)
65-74	1680	80.2 (79.3, 81.2)	222	12.3 (11.6, 13.0)	115	6.7 (6.1, 7.3)
75+	1312	78.5 (77.2, 79.8)	202	13.1 (12.0, 14.2)	90	6.7 (6.2, 7.3)
Gender						
Male	3893	82.5 (82.0, 83.1)	465	10.9 (10.5, 11.2)	221	5.9 (5.6, 6.1)
Female	2598	77.7 (77.0, 78.4)	419	14.4 (13.9, 14.9)	207	6.6 (6.2, 6.9)
Transgender	31	66.0 (56.5, 74.3)	8	30.8 (22.9, 40.1)	1	3.2 (2.6, 4.0)
Non-binary	52	91.5 (87.1, 94.5)	1	1.5 (0.2, 10.2)	1	5.7 (4.3, 7.5)
Other	15	89.9 (82.6, 94.4)	1	5.7 (3.2, 9.9)	1	4.4 (2.5, 7.7)
Race and Ethnicity						
White, Non-Hispanic	5043	82.7 (82.3, 83.1)	658	11.3 (11.0, 11.6)	274	5.1 (4.9, 5.3)
Black, Non-Hispanic	496	71.7 (69.8, 73.5)	77	16.0 (14.9, 17.2)	57	9.8 (8.8, 11.0)
Hispanic, any race	650	76.0 (74.5, 77.5)	114	15.9 (14.9, 17.0)	64	6.7 (6.2, 7.3)
American Indian or Alaska Native, Non-Hispanic	30	74.4 (68.0, 79.8)	9	12.3 (9.0, 16.7)	3	13.3 (10.7, 16.4)
Asian American or Pacific Islander, non-Hispanic	189	77.5 (74.6, 80.1)	26	13.6 (12.1, 15.3)	17	8.4 (7.2, 9.7)
Some other race, Non-Hispanic	15	100.0 (100.0, 100.0)	0	0.0 (0.0, 0.0)	0	0.0 (0.0, 0.0)
2+ Races, Non-Hispanic	194	84.3 (81.4, 86.9)	17	7.2 (6.0, 8.6)	20	8.2 (6.8, 9.7)

Table S5, continued.

Characteristics	The government should arrest reporters or journalists whose stories are critical of its policies or the President.					
	Do not agree		Somewhat agree		Strongly or very strongly agree	
	Unweighted n	Weighted % (95% CI)	Unweighted n	Weighted % (95% CI)	Unweighted n	Weighted % (95% CI)
Age						
18-24	99	73.4 (70.1, 76.5)	22	18.2 (16.0, 20.7)	8	6.6 (5.9, 7.5)
25-34	516	80.2 (78.8, 81.6)	65	13.7 (12.8, 14.7)	24	5.2 (4.6, 5.9)
35-44	779	79.9 (78.7, 81.0)	91	10.6 (10.0, 11.4)	64	7.5 (7.0, 8.1)
45-54	875	81.1 (80.0, 82.1)	89	9.4 (8.9, 9.9)	72	7.7 (7.1, 8.3)
55-64	1432	85.1 (84.4, 85.8)	123	9.3 (8.9, 9.8)	70	5.0 (4.6, 5.5)
65-74	1764	85.2 (84.5, 85.9)	154	8.3 (8.0, 8.6)	103	6.0 (5.5, 6.6)
75+	1389	83.5 (82.6, 84.3)	135	9.3 (8.7, 9.8)	85	6.4 (5.9, 6.8)
Gender						
Male	4017	84.2 (83.7, 84.6)	339	9.1 (8.9, 9.4)	222	5.8 (5.6, 6.0)
Female	2706	79.9 (79.3, 80.5)	328	11.9 (11.6, 12.3)	198	6.9 (6.6, 7.2)
Transgender	32	67.2 (57.9, 75.4)	4	24.3 (17.0, 33.4)	3	7.4 (6.0, 9.2)
Non-binary	52	91.5 (87.1, 94.5)	2	7.2 (4.5, 11.5)	0	0.0 (0.0, 0.0)
Other	16	94.3 (90.1, 96.8)	1	5.7 (3.2, 9.9)	0	0.0 (0.0, 0.0)
Race and Ethnicity						
White, Non-Hispanic	5258	85.8 (85.5, 86.1)	451	8.1 (7.9, 8.3)	271	5.3 (5.2, 5.5)
Black, Non-Hispanic	495	70.1 (68.1, 71.9)	83	18.4 (17.3, 19.5)	55	9.1 (8.1, 10.2)
Hispanic, any race	665	77.2 (75.8, 78.6)	92	14.0 (13.1, 14.9)	68	7.3 (6.7, 7.9)
American Indian or Alaska Native, Non-Hispanic	28	58.4 (49.2, 67.0)	9	25.3 (20.3, 31.1)	4	10.3 (7.2, 14.5)
Asian American or Pacific Islander, non-Hispanic	191	81.2 (78.8, 83.4)	25	10.8 (9.4, 12.4)	17	8.0 (7.1, 9.0)
Some other race, Non-Hispanic	15	100.0 (100.0, 100.0)	0	0.0 (0.0, 0.0)	0	0.0 (0.0, 0.0)
2+ Races, Non-Hispanic	202	86.6 (84.1, 88.8)	19	8.1 (6.8, 9.6)	11	5.1 (4.2, 6.1)

* Characteristics are as of participation in Wave 1 in 2022. Questions were asked of all participants.

Table S6. Sociodemographic characteristics and personal willingness to engage in violence in support of or opposition to the federal government*

Characteristics	In general, in a situation where you think force or violence is justified to advance an important political objective, how willing would you personally be to use force or violence this year…							
	To support the government's enforcement of its policies							
	Not asked the question		Not willling		Somewhat willing		Very or extremely willing	
	Unweighted n	Weighted % (95% CI)	Unweighted n	Weighted % (95% CI)	Unweighted n	Weighted % (95% CI)	Unweighted n	Weighted % (95% CI)
Age								
18-24	32	24.9 (20.4, 30.0)	80	59.3 (53.8, 64.5)	10	7.8 (5.6, 10.7)	7	5.9 (5.3, 6.7)
25-34	121	20.1 (17.4, 23.1)	430	69.2 (66.3, 71.9)	39	6.9 (6.3, 7.4)	13	2.4 (2.3, 2.6)
35-44	205	23.7 (21.5, 26.1)	633	64.6 (62.2, 66.9)	77	8.0 (7.4, 8.6)	20	2.5 (2.4, 2.6)
45-54	250	27.1 (24.9, 29.5)	704	64.9 (62.5, 67.2)	64	4.8 (4.1, 5.5)	21	1.9 (1.8, 2.0)
55-64	351	22.7 (20.9, 24.7)	1148	69.6 (67.7, 71.5)	108	5.9 (5.4, 6.3)	20	1.4 (1.3, 1.5)
65-74	409	20.9 (19.3, 22.5)	1450	71.3 (69.7, 72.9)	134	6.0 (5.5, 6.6)	25	1.2 (1.0, 1.3)
75+	244	15.5 (13.9, 17.3)	1198	74.8 (72.9, 76.6)	145	7.3 (6.6, 8.0)	16	1.3 (1.3, 1.4)
Gender								
Male	889	22.5 (21.4, 23.7)	3201	66.3 (65.1, 67.5)	406	7.8 (7.4, 8.2)	75	2.3 (2.2, 2.3)
Female	693	22.0 (20.9, 23.1)	2330	70.0 (68.8, 71.1)	165	5.4 (5.1, 5.7)	44	1.7 (1.6, 1.7)
Transgender	6	6.4 (2.7, 14.3)	29	68.7 (60.7, 75.8)	3	7.9 (6.3, 9.8)	2	17.0 (13.6, 21.0)
Non-binary	9	14.0 (7.3, 25.3)	44	79.0 (68.5, 86.7)	0	0.0 (0.0, 0.0)	1	5.7 (4.3, 7.5)
Other	5	20.8 (7.6, 45.8)	11	74.8 (50.6, 89.6)	1	4.4 (2.5, 7.7)	0	0.0 (0.0, 0.0)
Race and Ethnicity								
White, Non-Hispanic	1156	21.5 (20.7, 22.3)	4302	70.3 (69.4, 71.0)	445	6.3 (6.1, 6.5)	68	1.0 (1.0, 1.1)
Black, Non-Hispanic	134	20.7 (18.3, 23.3)	434	64.1 (61.4, 66.6)	44	8.6 (8.1, 9.1)	22	5.1 (4.8, 5.4)
Hispanic, any race	203	24.3 (21.9, 27.0)	556	64.7 (62.1, 67.3)	54	6.7 (5.8, 7.7)	17	2.8 (2.6, 2.9)
American Indian or Alaska Native, Non-Hispanic	6	22.9 (11.3, 41.1)	30	61.6 (48.7, 73.1)	4	3.8 (3.0, 4.6)	2	11.7 (9.4, 14.4)
Asian American or Pacific Islander, non-Hispanic	66	27.5 (22.4, 33.2)	149	65.2 (59.6, 70.4)	10	3.8 (2.8, 5.0)	6	2.4 (2.2, 2.8)
Some other race, Non-Hispanic	5	21.8 (12.2, 35.7)	8	61.5 (43.7, 76.7)	2	16.7 (10.7, 25.1)	0	0.0 (0.0, 0.0)
2+ Races, Non-Hispanic	42	14.7 (10.5, 20.1)	164	74.6 (68.7, 79.8)	18	6.8 (5.6, 8.2)	7	3.3 (2.8, 4.0)

Table S6, continued.

Characteristics	In general, in a situation where you think force or violence is justified to advance an important political objective, how willing would you personally be to use force or violence this year…							
	To oppose the government's enforcement of its policies							
	Not asked the question		Not willling		Somewhat willing		Very or extremely willing	
	Unweighted n	Weighted % (95% CI)	Unweighted n	Weighted % (95% CI)	Unweighted n	Weighted % (95% CI)	Unweighted n	Weighted % (95% CI)
Age								
18-24	32	24.9 (20.4, 30.0)	65	48.7 (42.9, 54.5)	23	17.2 (13.5, 21.8)	9	7.1 (5.4, 9.2)
25-34	121	20.1 (17.4, 23.1)	370	61.8 (58.7, 64.7)	78	10.9 (9.4, 12.6)	34	5.8 (5.1, 6.7)
35-44	205	23.7 (21.5, 26.1)	558	57.6 (55.1, 60.0)	139	14.1 (12.8, 15.4)	33	3.5 (3.2, 3.7)
45-54	250	27.1 (24.9, 29.5)	667	62.2 (59.7, 64.5)	105	8.0 (7.1, 9.1)	18	1.4 (1.3, 1.6)
55-64	351	22.7 (20.9, 24.7)	1113	67.7 (65.7, 69.7)	142	7.6 (6.5, 8.8)	20	1.5 (1.4, 1.7)
65-74	409	20.9 (19.3, 22.5)	1431	69.4 (67.8, 71.0)	158	8.0 (7.3, 8.8)	20	1.1 (1.0, 1.1)
75+	244	15.5 (13.9, 17.3)	1208	74.7 (72.8, 76.5)	143	7.7 (7.0, 8.5)	8	0.6 (0.6, 0.7)
Gender								
Male	889	22.5 (21.4, 23.7)	3103	63.0 (61.7, 64.1)	499	10.5 (9.9, 11.2)	82	2.9 (2.7, 3.1)
Female	693	22.0 (20.9, 23.1)	2233	66.2 (65.0, 67.5)	255	8.7 (8.0, 9.4)	49	2.0 (1.8, 2.2)
Transgender	6	6.4 (2.7, 14.3)	20	42.9 (32.0, 54.4)	11	31.5 (22.3, 42.4)	3	19.2 (15.2, 24.0)
Non-binary	9	14.0 (7.3, 25.3)	24	42.2 (28.9, 56.8)	15	26.8 (17.2, 39.4)	6	15.7 (9.4, 25.0)
Other	5	20.8 (7.6, 45.8)	6	25.6 (9.8, 52.0)	5	35.4 (14.7, 63.5)	1	18.2 (2.8, 63.0)
Race and Ethnicity								
White, Non-Hispanic	1156	21.5 (20.7, 22.3)	4146	66.4 (65.5, 67.2)	587	9.5 (9.1, 10.0)	82	1.7 (1.5, 1.9)
Black, Non-Hispanic	134	20.7 (18.3, 23.3)	407	59.8 (57.0, 62.5)	71	12.1 (10.7, 13.6)	22	5.7 (5.3, 6.2)
Hispanic, any race	203	24.3 (21.9, 27.0)	526	60.9 (58.2, 63.6)	71	8.7 (7.5, 10.1)	30	4.5 (4.0, 5.2)
American Indian or Alaska Native, Non-Hispanic	6	22.9 (11.3, 41.1)	30	50.7 (40.1, 61.3)	6	26.3 (20.8, 32.7)	0	0.0 (0.0, 0.0)
Asian American or Pacific Islander, non-Hispanic	66	27.5 (22.4, 33.2)	141	61.1 (55.2, 66.6)	21	8.3 (5.8, 11.7)	3	2.1 (1.9, 2.4)
Some other race, Non-Hispanic	5	21.8 (12.2, 35.7)	7	51.9 (32.1, 71.2)	3	26.3 (16.6, 39.0)	0	0.0 (0.0, 0.0)
2+ Races, Non-Hispanic	42	14.7 (10.5, 20.1)	155	68.0 (61.0, 74.2)	29	12.7 (9.3, 17.0)	5	4.1 (3.4, 4.8)

Table S6, continued.

Characteristics	In a situation where you think force or violence is justified to advance an important political objective, how willing would you personally be to use force or violence against a person because they are…							
	A person who speaks out against government policies or the President							
	Not asked the question		Not willling		Somewhat willing		Very or extremely willing	
	Unweighted n	Weighted % (95% CI)	Unweighted n	Weighted % (95% CI)	Unweighted n	Weighted % (95% CI)	Unweighted n	Weighted % (95% CI)
Age								
18-24	32	24.9 (20.4, 30.0)	84	61.7 (56.4, 66.7)	3	2.5 (2.2, 2.8)	9	7.8 (6.9, 8.7)
25-34	121	20.1 (17.4, 23.1)	443	70.8 (68.0, 73.6)	23	4.7 (4.4, 5.0)	18	3.4 (3.2, 3.6)
35-44	205	23.7 (21.5, 26.1)	660	67.2 (64.9, 69.5)	50	5.6 (5.3, 5.9)	19	2.1 (2.0, 2.2)
45-54	250	27.1 (24.9, 29.5)	755	68.5 (66.1, 70.7)	24	1.9 (1.8, 2.0)	14	1.7 (1.6, 1.8)
55-64	351	22.7 (20.9, 24.7)	1223	73.2 (71.2, 75.0)	45	2.8 (2.7, 3.0)	11	1.1 (1.0, 1.1)
65-74	409	20.9 (19.3, 22.5)	1549	75.3 (73.7, 76.8)	45	2.2 (2.2, 2.3)	19	1.1 (1.0, 1.1)
75+	244	15.5 (13.9, 17.3)	1310	80.0 (78.3, 81.7)	43	2.9 (2.8, 3.1)	10	0.8 (0.7, 0.8)
Gender								
Male	889	22.5 (21.4, 23.7)	3489	70.5 (69.4, 71.7)	143	3.9 (3.8, 3.9)	55	1.9 (1.9, 2.0)
Female	693	22.0 (20.9, 23.1)	2421	72.5 (71.3, 73.6)	85	2.8 (2.8, 2.9)	43	2.1 (2.0, 2.1)
Transgender	6	6.4 (2.7, 14.3)	30	71.5 (63.8, 78.2)	4	22.0 (17.6, 27.2)	0	0.0 (0.0, 0.0)
Non-binary	9	14.0 (7.3, 25.3)	43	78.3 (67.8, 86.1)	1	0.7 (0.5, 0.9)	1	5.7 (4.3, 7.5)
Other	5	20.8 (7.6, 45.8)	12	79.2 (54.2, 92.4)	0	0.0 (0.0, 0.0)	0	0.0 (0.0, 0.0)
Race and Ethnicity								
White, Non-Hispanic	1156	21.5 (20.7, 22.3)	4620	74.1 (73.3, 74.8)	158	2.8 (2.7, 2.8)	47	1.0 (0.9, 1.0)
Black, Non-Hispanic	134	20.7 (18.3, 23.3)	450	65.7 (63.1, 68.2)	29	6.2 (5.8, 6.5)	26	6.6 (6.2, 7.0)
Hispanic, any race	203	24.3 (21.9, 27.0)	582	68.7 (66.2, 71.2)	23	2.5 (2.4, 2.6)	21	3.0 (2.9, 3.2)
American Indian or Alaska Native, Non-Hispanic	6	22.9 (11.3, 41.1)	32	57.4 (45.5, 68.6)	3	15.0 (12.1, 18.5)	1	4.6 (3.7, 5.7)
Asian American or Pacific Islander, non-Hispanic	66	27.5 (22.4, 33.2)	153	65.5 (59.9, 70.7)	9	3.4 (3.0, 3.9)	3	1.7 (1.5, 1.9)
Some other race, Non-Hispanic	5	21.8 (12.2, 35.7)	9	63.7 (46.4, 78.1)	1	14.5 (9.3, 21.9)	0	0.0 (0.0, 0.0)
2+ Races, Non-Hispanic	42	14.7 (10.5, 20.1)	178	78.4 (72.8, 83.2)	10	5.6 (4.7, 6.6)	2	0.9 (0.8, 1.1)

Table S6, continued.

Characteristics	In a situation where you think force or violence is justified to advance an important political objective, how willing would you personally be to use force or violence against a person because they are…							
	A person who joins demonstrations against government policies or the President							
	Not asked the question		Not willling		Somewhat willing		Very or extremely willing	
	Unweighted n	Weighted % (95% CI)	Unweighted n	Weighted % (95% CI)	Unweighted n	Weighted % (95% CI)	Unweighted n	Weighted % (95% CI)
Age								
18-24	32	24.9 (20.4, 30.0)	82	60.4 (55.1, 65.5)	5	4.0 (3.5, 4.5)	9	7.5 (6.7, 8.5)
25-34	121	20.1 (17.4, 23.1)	445	71.6 (68.8, 74.4)	24	4.9 (4.6, 5.2)	15	2.4 (2.3, 2.6)
35-44	205	23.7 (21.5, 26.1)	652	66.0 (63.6, 68.3)	57	6.7 (6.3, 7.0)	20	2.2 (2.1, 2.4)
45-54	250	27.1 (24.9, 29.5)	735	67.0 (64.6, 69.3)	38	3.1 (2.9, 3.3)	20	2.0 (1.9, 2.1)
55-64	351	22.7 (20.9, 24.7)	1215	72.7 (70.8, 74.6)	53	3.5 (3.4, 3.7)	9	0.7 (0.7, 0.7)
65-74	409	20.9 (19.3, 22.5)	1530	73.7 (72.1, 75.3)	66	3.9 (3.6, 4.1)	16	0.9 (0.9, 1.0)
75+	244	15.5 (13.9, 17.3)	1269	78.1 (76.3, 79.8)	80	4.5 (4.3, 4.7)	10	1.0 (1.0, 1.1)
Gender								
Male	889	22.5 (21.4, 23.7)	3408	69.2 (68.0, 70.3)	223	5.4 (5.3, 5.6)	52	1.6 (1.6, 1.7)
Female	693	22.0 (20.9, 23.1)	2407	72.1 (71.0, 73.2)	94	3.3 (3.2, 3.3)	45	2.0 (2.0, 2.1)
Transgender	6	6.4 (2.7, 14.3)	30	71.5 (63.8, 78.2)	4	22.0 (17.6, 27.2)	0	0.0 (0.0, 0.0)
Non-binary	9	14.0 (7.3, 25.3)	44	79.0 (68.5, 86.7)	0	0.0 (0.0, 0.0)	1	5.7 (4.3, 7.5)
Other	5	20.8 (7.6, 45.8)	12	79.2 (54.2, 92.4)	0	0.0 (0.0, 0.0)	0	0.0 (0.0, 0.0)
Race and Ethnicity								
White, Non-Hispanic	1156	21.5 (20.7, 22.3)	4546	73.2 (72.4, 73.9)	224	3.5 (3.4, 3.6)	49	1.1 (1.0, 1.1)
Black, Non-Hispanic	134	20.7 (18.3, 23.3)	445	65.3 (62.7, 67.8)	38	8.0 (7.5, 8.5)	22	5.1 (4.8, 5.4)
Hispanic, any race	203	24.3 (21.9, 27.0)	570	67.5 (64.9, 69.9)	34	3.8 (3.6, 4.0)	21	2.9 (2.7, 3.0)
American Indian or Alaska Native, Non-Hispanic	6	22.9 (11.3, 41.1)	32	57.4 (45.5, 68.6)	4	19.6 (15.8, 24.1)	0	0.0 (0.0, 0.0)
Asian American or Pacific Islander, non-Hispanic	66	27.5 (22.4, 33.2)	148	63.0 (57.4, 68.3)	14	6.3 (5.6, 7.1)	3	1.3 (1.1, 1.4)
Some other race, Non-Hispanic	5	21.8 (12.2, 35.7)	9	63.7 (46.4, 78.1)	1	14.5 (9.3, 21.9)	0	0.0 (0.0, 0.0)
2+ Races, Non-Hispanic	42	14.7 (10.5, 20.1)	178	80.0 (74.5, 84.6)	8	1.4 (1.1, 1.8)	4	3.5 (2.9, 4.1)

Table S6, continued.

Characteristics	In a situation where you think force or violence is justified to advance an important political objective, how willing would you personally be to use force or violence against a person because they are… A reporter or journalist whose stories are critical of government policies or the President							
	Not asked the question		Not willling		Somewhat willing		Very or extremely willing	
	Unweighted n	Weighted % (95% CI)	Unweighted n	Weighted % (95% CI)	Unweighted n	Weighted % (95% CI)	Unweighted n	Weighted % (95% CI)
Age								
18-24	32	24.9 (20.4, 30.0)	86	63.7 (58.5, 68.7)	3	2.4 (2.1, 2.7)	7	5.8 (5.1, 6.5)
25-34	121	20.1 (17.4, 23.1)	447	71.6 (68.8, 74.4)	21	4.6 (4.3, 4.9)	14	2.3 (2.2, 2.5)
35-44	205	23.7 (21.5, 26.1)	657	66.9 (64.5, 69.2)	45	5.0 (4.7, 5.3)	27	3.0 (2.9, 3.2)
45-54	250	27.1 (24.9, 29.5)	753	68.2 (65.8, 70.4)	25	2.3 (2.2, 2.5)	15	1.6 (1.5, 1.7)
55-64	351	22.7 (20.9, 24.7)	1232	73.7 (71.7, 75.5)	31	1.9 (1.9, 2.0)	14	1.4 (1.3, 1.4)
65-74	409	20.9 (19.3, 22.5)	1538	74.7 (73.1, 76.3)	61	3.1 (3.0, 3.3)	13	0.7 (0.7, 0.7)
75+	244	15.5 (13.9, 17.3)	1306	79.6 (77.8, 81.2)	44	3.1 (2.9, 3.2)	11	1.1 (1.0, 1.1)
Gender								
Male	889	22.5 (21.4, 23.7)	3483	70.5 (69.4, 71.7)	140	3.7 (3.6, 3.8)	59	2.0 (1.9, 2.0)
Female	693	22.0 (20.9, 23.1)	2424	72.7 (71.6, 73.8)	86	2.8 (2.7, 2.9)	37	1.8 (1.7, 1.8)
Transgender	6	6.4 (2.7, 14.3)	29	70.4 (62.6, 77.2)	3	17.5 (14.0, 21.6)	2	5.7 (4.6, 7.0)
Non-binary	9	14.0 (7.3, 25.3)	44	79.0 (68.5, 86.7)	0	0.0 (0.0, 0.0)	1	5.7 (4.3, 7.5)
Other	5	20.8 (7.6, 45.8)	12	79.2 (54.2, 92.4)	0	0.0 (0.0, 0.0)	0	0.0 (0.0, 0.0)
Race and Ethnicity								
White, Non-Hispanic	1156	21.5 (20.7, 22.3)	4628	74.2 (73.4, 75.0)	151	2.7 (2.7, 2.8)	43	0.8 (0.8, 0.8)
Black, Non-Hispanic	134	20.7 (18.3, 23.3)	448	66.8 (64.2, 69.3)	30	6.3 (6.0, 6.7)	24	4.8 (4.6, 5.1)
Hispanic, any race	203	24.3 (21.9, 27.0)	571	67.9 (65.3, 70.3)	29	2.7 (2.6, 2.9)	25	3.6 (3.4, 3.8)
American Indian or Alaska Native, Non-Hispanic	6	22.9 (11.3, 41.1)	33	64.5 (50.9, 76.2)	0	0.0 (0.0, 0.0)	3	12.5 (10.1, 15.5)
Asian American or Pacific Islander, non-Hispanic	66	27.5 (22.4, 33.2)	150	64.3 (58.7, 69.5)	14	5.6 (5.0, 6.3)	1	0.8 (0.7, 0.9)
Some other race, Non-Hispanic	5	21.8 (12.2, 35.7)	9	63.7 (46.4, 78.1)	1	14.5 (9.3, 21.9)	0	0.0 (0.0, 0.0)
2+ Races, Non-Hispanic	42	14.7 (10.5, 20.1)	180	77.7 (72.0, 82.5)	5	2.2 (1.8, 2.6)	5	5.1 (4.3, 6.1)

Table S6, continued.

Characteristics	This question turns things around: In a situation where you think force or violence is justified to advance an important political objective, how willing would you personally be to use force or violence against a person because they are….							
	A person who speaks out in favor of government policies or the President							
	Not asked the question		Not willling		Somewhat willing		Very or extremely willing	
	Unweighted n	Weighted % (95% CI)	Unweighted n	Weighted % (95% CI)	Unweighted n	Weighted % (95% CI)	Unweighted n	Weighted % (95% CI)
Age								
18-24	32	24.9 (20.4, 30.0)	82	61.5 (56.1, 66.7)	10	7.7 (5.7, 10.4)	5	3.7 (3.3, 4.2)
25-34	121	20.1 (17.4, 23.1)	429	69.5 (66.6, 72.2)	36	6.0 (5.4, 6.6)	15	2.7 (2.6, 2.9)
35-44	205	23.7 (21.5, 26.1)	641	64.7 (62.4, 67.0)	59	6.8 (6.4, 7.3)	28	3.4 (3.2, 3.6)
45-54	250	27.1 (24.9, 29.5)	743	67.0 (64.6, 69.3)	31	3.0 (2.7, 3.3)	16	1.7 (1.7, 1.8)
55-64	351	22.7 (20.9, 24.7)	1229	73.3 (71.4, 75.2)	35	2.5 (2.2, 2.7)	13	1.1 (1.1, 1.2)
65-74	409	20.9 (19.3, 22.5)	1547	75.0 (73.4, 76.5)	50	2.8 (2.7, 2.9)	15	0.8 (0.8, 0.8)
75+	244	15.5 (13.9, 17.3)	1315	80.6 (78.8, 82.3)	31	1.6 (1.5, 1.8)	15	1.3 (1.3, 1.4)
Gender								
Male	889	22.5 (21.4, 23.7)	3486	70.5 (69.3, 71.6)	139	3.9 (3.6, 4.2)	60	2.0 (1.9, 2.0)
Female	693	22.0 (20.9, 23.1)	2397	71.1 (70.0, 72.2)	100	4.0 (3.9, 4.2)	43	1.9 (1.9, 2.0)
Transgender	6	6.4 (2.7, 14.3)	28	81.6 (72.2, 88.4)	5	10.5 (5.5, 19.0)	1	1.4 (1.2, 1.8)
Non-binary	9	14.0 (7.3, 25.3)	37	63.1 (50.6, 74.0)	7	19.7 (12.8, 29.0)	1	2.0 (1.5, 2.6)
Other	5	20.8 (7.6, 45.8)	11	73.1 (49.1, 88.5)	1	6.1 (3.4, 10.6)	0	0.0 (0.0, 0.0)
Race and Ethnicity								
White, Non-Hispanic	1156	21.5 (20.7, 22.3)	4616	73.6 (72.8, 74.4)	148	2.9 (2.7, 3.1)	56	1.1 (1.1, 1.2)
Black, Non-Hispanic	134	20.7 (18.3, 23.3)	443	65.4 (62.8, 67.9)	39	8.2 (7.7, 8.7)	19	4.5 (4.2, 4.7)
Hispanic, any race	203	24.3 (21.9, 27.0)	564	66.3 (63.7, 68.8)	39	4.7 (4.1, 5.4)	22	2.9 (2.8, 3.1)
American Indian or Alaska Native, Non-Hispanic	6	22.9 (11.3, 41.1)	34	65.4 (51.5, 77.1)	1	4.6 (3.7, 5.7)	1	7.1 (5.7, 8.8)
Asian American or Pacific Islander, non-Hispanic	66	27.5 (22.4, 33.2)	145	63.0 (57.3, 68.3)	15	6.5 (5.6, 7.4)	5	2.0 (1.8, 2.2)
Some other race, Non-Hispanic	5	21.8 (12.2, 35.7)	9	63.7 (46.4, 78.1)	1	14.5 (9.3, 21.9)	0	0.0 (0.0, 0.0)
2+ Races, Non-Hispanic	42	14.7 (10.5, 20.1)	175	74.5 (68.2, 79.9)	9	6.4 (4.2, 9.7)	4	3.4 (2.9, 4.0)

Table S6, continued.

Characteristics	This question turns things around: In a situation where you think force or violence is justified to advance an important political objective, how willing would you personally be to use force or violence against a person because they are….							
	A person who joins demonstrations in favor of government policies or the President							
	Not asked the question		Not willling		Somewhat willing		Very or extremely willing	
	Unweighted n	Weighted % (95% CI)	Unweighted n	Weighted % (95% CI)	Unweighted n	Weighted % (95% CI)	Unweighted n	Weighted % (95% CI)
Age								
18-24	32	24.9 (20.4, 30.0)	80	59.0 (53.5, 64.3)	12	10.3 (8.1, 12.9)	5	3.7 (3.3, 4.2)
25-34	121	20.1 (17.4, 23.1)	428	69.5 (66.6, 72.2)	40	6.9 (6.2, 7.6)	12	1.8 (1.7, 2.0)
35-44	205	23.7 (21.5, 26.1)	646	65.5 (63.1, 67.8)	57	6.5 (6.1, 7.1)	26	3.0 (2.9, 3.2)
45-54	250	27.1 (24.9, 29.5)	740	66.5 (64.1, 68.7)	30	2.8 (2.4, 3.2)	20	2.5 (2.4, 2.6)
55-64	351	22.7 (20.9, 24.7)	1222	72.8 (70.9, 74.6)	38	2.6 (2.4, 2.9)	14	1.2 (1.2, 1.3)
65-74	409	20.9 (19.3, 22.5)	1544	75.2 (73.6, 76.7)	54	2.7 (2.6, 2.9)	12	0.6 (0.6, 0.6)
75+	244	15.5 (13.9, 17.3)	1318	80.8 (79.0, 82.5)	34	2.1 (1.9, 2.3)	9	0.7 (0.7, 0.7)
Gender								
Male	889	22.5 (21.4, 23.7)	3482	70.2 (69.1, 71.4)	141	4.1 (3.8, 4.4)	56	1.9 (1.9, 2.0)
Female	693	22.0 (20.9, 23.1)	2397	71.3 (70.2, 72.4)	108	4.3 (4.1, 4.5)	37	1.5 (1.5, 1.6)
Transgender	6	6.4 (2.7, 14.3)	25	57.9 (47.7, 67.5)	9	35.7 (27.3, 45.1)	0	0.0 (0.0, 0.0)
Non-binary	9	14.0 (7.3, 25.3)	36	61.4 (48.9, 72.6)	6	14.1 (8.1, 23.5)	3	9.2 (6.9, 12.0)
Other	5	20.8 (7.6, 45.8)	11	73.1 (49.1, 88.5)	1	6.1 (3.4, 10.6)	0	0.0 (0.0, 0.0)
Race and Ethnicity								
White, Non-Hispanic	1156	21.5 (20.7, 22.3)	4608	73.5 (72.7, 74.3)	159	3.1 (2.9, 3.3)	51	1.0 (1.0, 1.0)
Black, Non-Hispanic	134	20.7 (18.3, 23.3)	439	64.3 (61.6, 66.8)	43	9.8 (9.2, 10.5)	19	4.0 (3.8, 4.2)
Hispanic, any race	203	24.3 (21.9, 27.0)	567	66.9 (64.3, 69.4)	37	4.4 (3.8, 5.1)	20	2.6 (2.4, 2.7)
American Indian or Alaska Native, Non-Hispanic	6	22.9 (11.3, 41.1)	32	58.8 (46.5, 70.0)	2	6.6 (5.4, 8.2)	2	11.7 (9.4, 14.4)
Asian American or Pacific Islander, non-Hispanic	66	27.5 (22.4, 33.2)	148	63.8 (58.2, 69.1)	14	5.9 (5.1, 6.8)	3	1.7 (1.5, 1.9)
Some other race, Non-Hispanic	5	21.8 (12.2, 35.7)	9	63.7 (46.4, 78.1)	1	14.5 (9.3, 21.9)	0	0.0 (0.0, 0.0)
2+ Races, Non-Hispanic	42	14.7 (10.5, 20.1)	175	73.6 (67.3, 79.1)	9	6.5 (4.2, 9.8)	3	3.2 (2.7, 3.9)

Table S6, continued.

Characteristics	This question turns things around: In a situation where you think force or violence is justified to advance an important political objective, how willing would you personally be to use force or violence against a person because they are....							
	A reporter or journalist whose stories support government policies or the President							
	Not asked the question		Not willling		Somewhat willing		Very or extremely willing	
	Unweighted n	Weighted % (95% CI)	Unweighted n	Weighted % (95% CI)	Unweighted n	Weighted % (95% CI)	Unweighted n	Weighted % (95% CI)
Age								
18-24	32	24.9 (20.4, 30.0)	85	63.2 (57.8, 68.2)	7	6.0 (4.4, 8.1)	5	3.8 (3.4, 4.3)
25-34	121	20.1 (17.4, 23.1)	439	70.8 (67.9, 73.5)	25	4.4 (3.9, 4.9)	16	3.0 (2.8, 3.2)
35-44	205	23.7 (21.5, 26.1)	651	66.0 (63.7, 68.3)	56	6.6 (6.1, 7.1)	22	2.4 (2.3, 2.6)
45-54	250	27.1 (24.9, 29.5)	748	67.3 (65.0, 69.6)	24	2.3 (2.0, 2.6)	19	2.2 (2.1, 2.3)
55-64	351	22.7 (20.9, 24.7)	1233	73.2 (71.2, 75.0)	32	2.4 (2.2, 2.6)	11	1.3 (1.2, 1.3)
65-74	409	20.9 (19.3, 22.5)	1550	74.8 (73.2, 76.3)	48	3.1 (2.9, 3.2)	13	0.7 (0.6, 0.7)
75+	244	15.5 (13.9, 17.3)	1322	80.9 (79.1, 82.6)	31	2.0 (1.8, 2.2)	11	0.8 (0.8, 0.9)
Gender								
Male	889	22.5 (21.4, 23.7)	3504	70.6 (69.5, 71.8)	119	3.2 (3.0, 3.5)	62	2.5 (2.5, 2.6)
Female	693	22.0 (20.9, 23.1)	2417	72.0 (70.8, 73.1)	95	3.9 (3.8, 4.1)	31	1.2 (1.2, 1.2)
Transgender	6	6.4 (2.7, 14.3)	30	86.1 (78.9, 91.1)	3	3.9 (1.9, 7.8)	1	3.5 (2.8, 4.4)
Non-binary	9	14.0 (7.3, 25.3)	38	66.2 (53.9, 76.7)	5	10.8 (5.3, 20.8)	2	7.6 (5.8, 10.0)
Other	5	20.8 (7.6, 45.8)	12	79.2 (54.2, 92.4)	0	0.0 (0.0, 0.0)	0	0.0 (0.0, 0.0)
Race and Ethnicity								
White, Non-Hispanic	1156	21.5 (20.7, 22.3)	4643	74.3 (73.5, 75.0)	131	2.6 (2.4, 2.8)	47	0.8 (0.7, 0.8)
Black, Non-Hispanic	134	20.7 (18.3, 23.3)	447	65.1 (62.5, 67.7)	33	7.8 (7.4, 8.3)	21	5.1 (4.8, 5.4)
Hispanic, any race	203	24.3 (21.9, 27.0)	566	66.6 (64.0, 69.1)	40	4.7 (4.2, 5.2)	20	2.8 (2.7, 3.0)
American Indian or Alaska Native, Non-Hispanic	6	22.9 (11.3, 41.1)	34	65.4 (51.5, 77.1)	0	0.0 (0.0, 0.0)	2	11.7 (9.4, 14.4)
Asian American or Pacific Islander, non-Hispanic	66	27.5 (22.4, 33.2)	150	64.5 (58.9, 69.8)	11	4.2 (3.5, 4.9)	4	2.7 (2.4, 3.0)
Some other race, Non-Hispanic	5	21.8 (12.2, 35.7)	9	63.7 (46.4, 78.1)	1	14.5 (9.3, 21.9)	0	0.0 (0.0, 0.0)
2+ Races, Non-Hispanic	42	14.7 (10.5, 20.1)	179	77.0 (70.8, 82.2)	7	2.8 (1.1, 7.0)	3	4.9 (4.1, 5.8)

* Characteristics are as of participation in Wave 1 in 2022. Respondents who did not answer the question "In general...to advance an important political objective that you support" (n = 57) or who answered “never justified” to all prior questions on the use of force or violence to advance political objectives were not asked questions on their personal willingness to use political violence.

Table S7. Party/MAGA affiliation and views on democracy and authoritarianism

The United States as you see it now, in 2025	Population Estimates by Party Category for 2025*							
	MAGA Affiliation						No MAGA Affiliation	
	MAGA Republican (n = 1536)		MAGA Supporter, Republican (n = 694)		MAGA Supporter, Non-Republican (n = 194)		Non-MAGA, strong Republican (n = 304)	
	Unweighted n	Weighted % (95% CI)	Unweighted n	Weighted % (95% CI)	Unweighted n	Weighted % (95% CI)	Unweighted n	Weighted % (95% CI)
How important do you think it is for the United States to remain a democracy?								
Not important	55	4.2 (3.0, 5.9)	18	2.8 (1.6, 5.1)	13	9.5 (4.1, 20.5)	11	7.0 (3.5, 13.7)
Somewhat important	77	8.0 (6.1, 10.5)	28	6.0 (3.7, 9.4)	13	5.5 (2.5, 12.0)	13	8.1 (4.2, 15.1)
Very or extremely important	1380	86.4 (83.6, 88.8)	645	90.8 (87.1, 93.5)	166	84.5 (74.1, 91.3)	277	83.7 (75.7, 89.4)
Non-response	24	1.4 (0.9, 2.1)	3	0.4 (0.1, 1.3)	2	0.4 (0.1, 1.7)	3	1.2 (0.3, 4.6)
aPD; q-value†	-12.53 (-15.53, -9.53); <0.0001		-9.51 (-13.06, -5.95); <0.0001		-3.91 (-10.54, 2.71); 1.00		-13.97 (-20.9, -7.04); 0.002	
When thinking about democracy in the United States these days, do you believe…								
There is a serious threat to our democracy	681	43.5 (40.1, 46.8)	241	32.0 (27.5, 36.8)	81	43.2 (33.6, 53.3)	123	33.8 (27.3, 41.0)
There may be a threat to our democracy, but it is not serious	437	31.3 (28.2, 34.6)	259	40.6 (35.5, 46.0)	57	31.5 (22.7, 41.8)	119	46.6 (38.9, 54.5)
There is no threat to our democracy.	395	23.4 (20.7, 26.3)	187	25.5 (21.4, 30.0)	54	24.7 (16.8, 34.6)	61	18.3 (13.4, 24.5)
Non-response	23	1.9 (1.2, 3.0)	7	1.9 (0.7, 5.2)	2	0.7 (0.1, 3.1)	1	1.3 (0.2, 8.5)
aPD; q-value†	-50.05 (-54.48, -45.62); <0.0001		-61.17 (-66.49, -55.86); <0.0001		-43.38 (-53.31, -33.44); <0.0001		-58.56 (-65.7, -51.43); <0.0001	
How much do you agree or disagree with the following statements about democracy in the United States?								
Democracy is the best form of government.								
Do not agree	137	9.2 (7.5, 11.3)	58	6.6 (4.7, 9.0)	19	10.2 (4.9, 20.2)	23	10.6 (6.3, 17.5)
Somewhat agree	243	20.4 (17.5, 23.7)	124	21.0 (17.0, 25.6)	46	27.7 (19.3, 37.9)	50	23.9 (17.3, 32.0)
Strongly or very strongly agree	1142	69.2 (65.8, 72.4)	510	72.0 (67.2, 76.3)	127	60.1 (49.5, 69.8)	230	65.2 (56.9, 72.7)
Non-response	14	1.1 (0.6, 2.2)	2	0.5 (0.1, 2.4)	2	2.0 (0.3, 11.3)	1	0.3 (0.0, 2.0)
aPD; q-value†	-17.66 (-22.08, -13.24); <0.0001		-16.03 (-21.72, -10.33); <0.0001		-14.24 (-24.3, -4.19); 0.08		-18.81 (-26.99, -10.63); 0.0002	
Having a strong leader for America is more important than having a democracy.								
Do not agree	736	40.8 (37.6, 44.1)	421	57.3 (52.0, 62.4)	89	40.2 (30.8, 50.3)	171	46.4 (38.9, 54.0)
Somewhat agree	343	25.2 (22.3, 28.4)	153	24.1 (19.9, 28.9)	48	26.5 (18.5, 36.4)	59	23.7 (17.5, 31.3)
Strongly or very strongly agree	431	32.1 (28.9, 35.4)	114	17.6 (13.7, 22.3)	55	30.2 (21.6, 40.3)	73	29.6 (22.6, 37.8)
Non-response	26	1.9 (1.2, 3.0)	6	1.0 (0.4, 2.6)	2	3.2 (0.8, 12.0)	1	0.3 (0.0, 2.0)
aPD; q-value†	26.38 (22.24, 30.52); <0.0001		12.03 (7.1, 16.95); <0.0001		18.92 (9.83, 28.02); 0.001		22.99 (14.82, 31.15); <0.0001	
We should suspend Congress for a few years so a strong leader can clean up the mess made by politicians in Washington.								
Do not agree	924	53.7 (50.3, 57.0)	527	71.7 (66.6, 76.3)	108	42.0 (32.7, 51.8)	205	56.8 (48.7, 64.6)
Somewhat agree	309	23.1 (20.3, 26.2)	112	18.0 (14.4, 22.4)	36	23.5 (15.7, 33.8)	60	24.8 (18.0, 33.1)
Strongly or very strongly agree	286	21.7 (19.0, 24.7)	54	9.9 (6.9, 14.0)	49	32.6 (23.6, 43.2)	37	16.9 (11.5, 24.3)
Non-response	17	1.5 (0.8, 2.6)	1	0.4 (0.1, 2.7)	1	1.9 (0.3, 12.0)	2	1.5 (0.3, 7.1)
aPD; q-value†	14.42 (10.73, 18.1); <0.0001		3.93 (-0.23, 8.09); 0.66		21.35 (11.44, 31.26); 0.0006		9.56 (2.61, 16.51); 0.10	
Which is more important to you…?								
Having election outcomes determined democratically	806	48.8 (45.5, 52.2)	426	59.6 (54.4, 64.6)	102	49.6 (39.6, 59.7)	179	48.6 (41.0, 56.3)
Having political leaders I can trust to look out for my values and interests	684	47.1 (43.8, 50.5)	253	37.3 (32.4, 42.4)	80	43.7 (34.1, 53.9)	120	48.6 (40.9, 56.4)
Non-response	46	4.1 (2.7, 6.0)	15	3.1 (1.4, 6.7)	12	6.7 (2.8, 14.9)	5	2.8 (0.9, 8.4)
aPD; q-value†	-30.8 (-35.53, -26.06); <0.0001		-22.67 (-28.73, -16.6); <0.0001		-21.42 (-32.97, -9.86); 0.005		-29.34 (-37.28, -21.39); <0.0001	

Table S7, continued.

The United States as you see it now, in 2025	Population Estimates by Party Category for 2025*							
	No MAGA Affiliation							
	Non-MAGA, leans or not strong Republican (n = 1003)		Non-MAGA, Independent (n = 960)		Non-MAGA, leans or not strong Democrat (n = 1940)		Non-MAGA, strong Democrat (n = 1405)	
	Unweighted n	Weighted % (95% CI)	Unweighted n	Weighted % (95% CI)	Unweighted n	Weighted % (95% CI)	Unweighted n	Weighted % (95% CI)
How important do you think it is for the United States to remain a democracy?								
Not important	19	2.6 (1.5, 4.4)	55	9.1 (6.8, 12.2)	4	0.2 (0.1, 0.8)	4	0.6 (0.2, 1.7)
Somewhat important	56	7.6 (5.6, 10.2)	94	14.8 (11.8, 18.5)	60	6.3 (4.7, 8.4)	10	1.2 (0.6, 2.3)
Very or extremely important	920	89.1 (86.1, 91.5)	801	74.9 (70.7, 78.6)	1871	93.1 (90.9, 94.7)	1391	98.2 (96.9, 99.0)
Non-response	8	0.8 (0.3, 1.8)	10	1.2 (0.5, 2.5)	5	0.4 (0.1, 1.2)	0	0.0 (0.0, 0.0)
aPD; q-value†	-9.57 (-12.6, -6.53); <0.0001		-18.27 (-22.12, -14.42); <0.0001		-4.33 (-6.57, -2.08); 0.003		Reference	
When thinking about democracy in the United States these days, do you believe…								
There is a serious threat to our democracy	422	38.9 (35.0, 43.0)	632	60.4 (56.2, 64.6)	1641	78.2 (75.4, 80.8)	1326	90.8 (88.1, 93.0)
There may be a threat to our democracy, but it is not serious	421	44.5 (40.4, 48.7)	236	27.4 (23.7, 31.4)	246	17.4 (15.0, 20.0)	59	6.2 (4.5, 8.5)
There is no threat to our democracy.	149	15.4 (12.6, 18.7)	71	8.7 (6.5, 11.5)	49	3.9 (2.8, 5.5)	17	2.8 (1.6, 4.7)
Non-response	11	1.2 (0.6, 2.4)	21	3.5 (2.1, 5.8)	4	0.5 (0.2, 1.4)	3	0.2 (0.1, 0.8)
aPD; q-value†	-51.72 (-56.46, -46.98); <0.0001		-25.26 (-30.16, -20.36); <0.0001		-9.81 (-13.31, -6.31); <0.0001		Reference	
How much do you agree or disagree with the following statements about democracy in the United States?								
Democracy is the best form of government.								
Do not agree	62	8.0 (5.9, 10.7)	96	15.9 (12.7, 19.7)	40	2.6 (1.7, 3.8)	18	2.1 (1.2, 3.7)
Somewhat agree	195	23.8 (20.3, 27.7)	243	29.8 (26.1, 33.9)	321	22.5 (19.9, 25.3)	145	13.6 (11.2, 16.4)
Strongly or very strongly agree	737	67.1 (63.0, 71.0)	598	50.6 (46.4, 54.8)	1574	74.5 (71.6, 77.2)	1238	83.8 (80.8, 86.5)
Non-response	9	1.0 (0.5, 2.3)	23	3.7 (2.3, 6.0)	5	0.5 (0.2, 1.4)	4	0.5 (0.2, 1.4)
aPD; q-value†	-17.18 (-22.01, -12.35); <0.0001		-23.78 (-28.77, -18.79); <0.0001		-7.07 (-10.89, -3.26); 0.005		Reference	
Having a strong leader for America is more important than having a democracy.								
Do not agree	687	63.5 (59.3, 67.4)	670	63.0 (58.8, 67.1)	1624	78.2 (75.4, 80.8)	1223	84.3 (81.4, 86.9)
Somewhat agree	196	22.5 (19.1, 26.3)	158	19.5 (16.3, 23.1)	194	13.3 (11.2, 15.7)	90	7.6 (5.8, 9.9)
Strongly or very strongly agree	108	12.8 (10.2, 15.9)	114	14.6 (11.7, 18.0)	113	7.8 (6.2, 9.8)	88	7.5 (5.8, 9.7)
Non-response	12	1.2 (0.6, 2.5)	18	2.9 (1.7, 5.1)	9	0.8 (0.4, 1.6)	4	0.5 (0.2, 1.6)
aPD; q-value†	7.66 (3.94, 11.39); 0.0013		4.72 (1.01, 8.43); 0.17		0.33 (-2.4, 3.05); 1.00		Reference	
We should suspend Congress for a few years so a strong leader can clean up the mess made by politicians in Washington.								
Do not agree	768	69.7 (65.6, 73.5)	713	68.5 (64.3, 72.4)	1651	80.0 (77.3, 82.5)	1194	80.5 (77.4, 83.3)
Somewhat agree	144	17.9 (14.8, 21.5)	124	16.2 (13.2, 19.8)	173	12.0 (10.1, 14.3)	107	11.1 (8.8, 13.7)
Strongly or very strongly agree	79	10.4 (8.1, 13.2)	102	11.9 (9.4, 14.9)	106	6.8 (5.3, 8.8)	93	7.5 (5.9, 9.6)
Non-response	12	2.0 (1.0, 4.1)	21	3.4 (2.0, 5.6)	10	1.1 (0.5, 2.1)	11	0.9 (0.4, 1.9)
aPD; q-value†	4.41 (0.98, 7.85); 0.16		1.88 (-1.6, 5.36); 1.00		-0.46 (-3.1, 2.18); 1.00		Reference	
Which is more important to you…?								
Having election outcomes determined democratically	651	58.1 (53.9, 62.2)	589	51.8 (47.5, 56.0)	1497	69.4 (66.3, 72.2)	1134	75.6 (72.3, 78.6)
Having political leaders I can trust to look out for my values and interests	328	37.5 (33.5, 41.6)	307	37.0 (33.0, 41.2)	406	27.6 (24.8, 30.5)	233	19.7 (17.0, 22.8)
Non-response	24	4.4 (2.7, 7.2)	64	11.2 (8.5, 14.7)	37	3.1 (2.0, 4.6)	38	4.7 (3.1, 6.9)
aPD; q-value†	-21.03 (-26.06, -16); <0.0001		-16.52 (-21.67, -11.38); <0.0001		-7.57 (-11.53, -3.61); 0.004		Reference	

* Questions were asked of all participants.

† Adjusted prevalence differences (aPDs) are absolute percentage point (pp) differences for “very or extremely important” responses for item 1, “there is a serious threat to our democracy” for item 2, “strongly or very strongly agree” responses for items 3-5, and “having election outcomes determined democratically” responses for item 6. They are adjusted for age, gender, race and ethnicity, education, income, Census division, marital status, homeownership, rurality, firearm ownership, alcohol consumption, military service, and history of non-traffic arrest. Q-values, also known as FDR-adjusted (or FDR-corrected) p-values, were calculated using the Benjamini-Yekutieli method [7]. Q-values represent the probability that the given difference would be a false discovery; they represent the expected proportion of “false positives” that would be seen among the collection of all differences whose q-values were at or below the given q-value. Item non-responses are not reported in the tables but are included in the prevalence calculations.

Table S8. Party/MAGA affiliation and support for violence initiated by the federal government

Actions the federal government might take this year	Population Estimates by Party Category for 2025*							
	MAGA Affiliation						No MAGA Affiliation	
	MAGA Republican (n = 1536)		MAGA Supporter, Republican (n = 694)		MAGA Supporter, Non-Republican (n = 194)		Non-MAGA, strong Republican (n = 304)	
	Unweighted n	Weighted % (95% CI)	Unweighted n	Weighted % (95% CI)	Unweighted n	Weighted % (95% CI)	Unweighted n	Weighted % (95% CI)
How much do you agree or disagree with the following statements?								
The government should use the military to help enforce its policies in the United States.								
Do not agree	575	35.9 (32.7, 39.2)	339	46.1 (40.9, 51.3)	85	38.1 (29.0, 48.1)	177	53.8 (45.9, 61.4)
Somewhat agree	607	39.1 (35.8, 42.4)	256	38.2 (33.2, 43.5)	67	36.8 (27.6, 47.0)	87	30.5 (23.9, 38.2)
Strongly or very strongly agree	338	23.6 (20.9, 26.7)	94	14.7 (11.2, 19.1)	39	22.4 (14.6, 32.7)	39	15.4 (10.2, 22.6)
Non-response	16	1.4 (0.8, 2.4)	5	1.0 (0.4, 2.7)	3	2.8 (0.7, 10.5)	1	0.3 (0.0, 2.0)
aPD; q-value†	22.16 (18.51, 25.8); <0.0001		13.65 (9.09, 18.21); <0.0001		15.42 (6.9, 23.94); 0.007		12.22 (5.96, 18.49); 0.003	
The government should use private armed militia groups to help enforce its policies in the United States.								
Do not agree	1206	73.0 (69.7, 76.1)	602	83.8 (79.4, 87.4)	132	55.1 (44.7, 65.1)	245	73.1 (64.9, 80.0)
Somewhat agree	208	16.0 (13.5, 18.9)	62	10.1 (7.5, 13.6)	34	25.2 (17.0, 35.6)	43	18.1 (12.6, 25.4)
Strongly or very strongly agree	107	9.6 (7.6, 12.2)	23	4.7 (2.6, 8.3)	24	16.0 (9.3, 26.1)	15	8.4 (4.3, 16.0)
Non-response	15	1.4 (0.8, 2.4)	7	1.4 (0.6, 3.1)	4	3.7 (1.2, 11.0)	1	0.3 (0.0, 2.2)
aPD; q-value†	9.6 (6.82, 12.38); <0.0001		4.8 (1.44, 8.16); 0.08		10.6 (3.19, 18.01); 0.08		7.13 (1.44, 12.82); 0.18	
And how much do you agree or disagree with these statements? The government should arrest…								
People who speak out against its policies or the President								
Do not agree	1248	79.0 (75.9, 81.8)	619	89.2 (85.7, 92.0)	131	58.5 (48.1, 68.2)	246	78.1 (70.5, 84.2)
Somewhat agree	192	14.4 (12.0, 17.1)	56	8.6 (6.1, 12.0)	36	25.5 (17.4, 35.7)	37	13.3 (8.5, 20.1)
Strongly or very strongly agree	87	6.1 (4.6, 8.0)	14	1.7 (0.9, 3.4)	24	14.9 (8.7, 24.3)	18	8.1 (4.6, 14.0)
Non-response	9	0.6 (0.2, 1.3)	5	0.5 (0.2, 1.2)	3	1.1 (0.3, 3.6)	3	0.5 (0.2, 1.8)
aPD; q-value†	0.61 (-2.13, 3.35); 1.00		-3.66 (-6.02, -1.3); 0.04		7.82 (-0.39, 16.03); 0.66		1.36 (-3.23, 5.94); 1.00	
People who join demonstrations against its policies or the President								
Do not agree	1057	66.5 (63.1, 69.7)	560	79.2 (74.5, 83.2)	124	57.5 (47.2, 67.3)	234	72.7 (64.8, 79.4)
Somewhat agree	332	23.2 (20.3, 26.3)	102	16.2 (12.5, 20.8)	40	22.2 (14.9, 31.7)	47	16.4 (11.5, 22.9)
Strongly or very strongly agree	133	9.3 (7.5, 11.4)	27	3.9 (2.5, 6.1)	27	19.2 (12.0, 29.4)	22	10.8 (6.2, 18.2)
Non-response	14	1.0 (0.5, 1.9)	5	0.7 (0.2, 1.9)	3	1.1 (0.3, 3.6)	1	0.1 (0.0, 0.6)
aPD; q-value†	4.14 (1.25, 7.02); 0.08		-0.99 (-3.69, 1.7); 1.00		12.37 (3.18, 21.56); 0.12		4.4 (-1.48, 10.29); 1.00	
Reporters and journalists whose stories are critical of its policies or the President								
Do not agree	1184	74.9 (71.7, 77.7)	595	84.9 (81.0, 88.2)	130	57.6 (47.2, 67.3)	226	65.0 (56.7, 72.5)
Somewhat agree	203	14.5 (12.1, 17.2)	76	10.9 (8.2, 14.4)	37	24.6 (16.8, 34.6)	50	20.3 (14.5, 27.7)
Strongly or very strongly agree	135	9.7 (7.9, 11.9)	21	3.7 (2.1, 6.4)	25	17.2 (10.5, 26.9)	26	14.5 (9.0, 22.7)
Non-response	14	0.9 (0.5, 1.8)	2	0.4 (0.1, 1.9)	2	0.6 (0.1, 2.5)	2	0.2 (0.1, 0.9)
aPD; q-value†	4.53 (1.58, 7.48); 0.05		-1.78 (-4.45, 0.89); 1.00		8.5 (0.55, 16.45); 0.40		8.19 (1.3, 15.08); 0.23	

Table S8, continued.

Actions the federal government might take this year	Population Estimates by Party Category for 2025*							
	No MAGA Affiliation							
	Non-MAGA, leans or not strong Republican (n = 1003)		Non-MAGA, Independent (n = 960)		Non-MAGA, leans or not strong Democrat (n = 1940)		Non-MAGA, strong Democrat (n = 1405)	
	Unweighted n	Weighted % (95% CI)	Unweighted n	Weighted % (95% CI)	Unweighted n	Weighted % (95% CI)	Unweighted n	Weighted % (95% CI)
How much do you agree or disagree with the following statements?								
The government should use the military to help enforce its policies in the United States.								
Do not agree	624	58.8 (54.6, 62.8)	703	68.0 (63.8, 72.0)	1649	82.6 (80.1, 84.8)	1257	86.3 (83.5, 88.7)
Somewhat agree	282	28.4 (24.8, 32.2)	190	24.1 (20.5, 28.1)	231	13.3 (11.3, 15.5)	101	8.8 (6.9, 11.1)
Strongly or very strongly agree	85	11.2 (8.6, 14.4)	55	6.3 (4.5, 8.6)	53	3.7 (2.5, 5.3)	39	4.1 (2.7, 6.1)
Non-response	12	1.7 (0.8, 3.4)	12	1.7 (0.8, 3.3)	7	0.5 (0.2, 1.1)	8	0.9 (0.4, 1.9)
aPD; q-value†	9.41 (6.04, 12.78); <0.0001		1.42 (-1.43, 4.27); 1.00		-0.41 (-2.57, 1.75); 1.00		Reference	
The government should use private armed militia groups to help enforce its policies in the United States.								
Do not agree	898	85.4 (81.8, 88.4)	849	84.3 (80.6, 87.3)	1840	91.8 (89.7, 93.5)	1349	92.4 (89.7, 94.4)
Somewhat agree	70	8.8 (6.6, 11.6)	75	10.5 (8.0, 13.6)	67	5.5 (4.1, 7.3)	36	4.5 (3.0, 6.7)
Strongly or very strongly agree	26	4.4 (2.7, 7.2)	23	3.3 (1.9, 5.5)	27	2.3 (1.4, 3.7)	11	1.9 (1.0, 3.7)
Non-response	9	1.4 (0.7, 2.9)	13	2.0 (1.0, 3.8)	6	0.4 (0.2, 1.0)	9	1.3 (0.6, 2.7)
aPD; q-value†	3.49 (1.04, 5.94); 0.08		-0.11 (-2.41, 2.18); 1.00		-0.11 (-1.84, 1.63); 1.00		Reference	
And how much do you agree or disagree with these statements? The government should arrest…								
People who speak out against its policies or the President								
Do not agree	889	85.0 (81.5, 88.0)	834	81.4 (77.7, 84.7)	1773	88.1 (85.7, 90.1)	1274	87.9 (85.2, 90.1)
Somewhat agree	79	10.2 (7.7, 13.4)	68	9.8 (7.5, 12.8)	79	6.3 (4.8, 8.3)	44	4.1 (2.9, 5.9)
Strongly or very strongly agree	29	4.0 (2.6, 6.1)	40	6.0 (4.2, 8.5)	74	4.4 (3.3, 5.8)	77	6.9 (5.2, 9.1)
Non-response	6	0.8 (0.3, 2.0)	18	2.7 (1.5, 4.8)	14	1.2 (0.6, 2.3)	10	1.1 (0.5, 2.1)
aPD; q-value†	-1.79 (-4.5, 0.93); 1.00		-2.18 (-5.13, 0.78); 1.00		-2.33 (-4.67, 0.01); 0.56		Reference	
People who join demonstrations against its policies or the President								
Do not agree	835	79.8 (76.0, 83.2)	798	78.7 (75.0, 82.0)	1753	86.6 (84.2, 88.8)	1269	87.3 (84.7, 89.6)
Somewhat agree	123	13.9 (11.1, 17.2)	106	13.7 (11.0, 16.9)	100	8.2 (6.4, 10.4)	51	5.3 (3.8, 7.3)
Strongly or very strongly agree	38	5.4 (3.5, 8.1)	35	4.6 (3.1, 6.7)	77	4.3 (3.3, 5.7)	76	6.4 (4.8, 8.5)
Non-response	7	0.9 (0.4, 2.2)	21	3.0 (1.8, 5.1)	10	0.9 (0.4, 1.8)	9	1.0 (0.5, 2.0)
aPD; q-value†	0.34 (-2.56, 3.24); 1.00		-3.01 (-5.66, -0.36); 0.29		-1.94 (-4.16, 0.29); 0.86		Reference	
Reporters and journalists whose stories are critical of its policies or the President								
Do not agree	862	82.3 (78.6, 85.4)	815	79.6 (75.8, 83.0)	1779	87.5 (85.0, 89.6)	1275	87.4 (84.7, 89.7)
Somewhat agree	98	11.6 (9.0, 14.8)	89	12.2 (9.5, 15.3)	82	7.9 (6.1, 10.2)	45	4.9 (3.4, 7.0)
Strongly or very strongly agree	37	5.3 (3.5, 7.8)	37	4.9 (3.3, 7.1)	69	3.8 (2.8, 5.1)	76	6.5 (4.9, 8.5)
Non-response	6	0.9 (0.4, 2.2)	19	3.3 (1.9, 5.7)	10	0.8 (0.4, 1.7)	9	1.2 (0.6, 2.4)
aPD; q-value†	0.01 (-2.85, 2.87); 1.00		-2.51 (-5.26, 0.24); 0.73		-2.44 (-4.61, -0.26); 0.31		Reference	

* Questions were asked of all participants.

† Adjusted prevalence differences (aPDs) are absolute percentage point (pp) differences for "strongly or very strongly agree" responses. They are adjusted for age, gender, race and ethnicity, education, income, Census division, marital status, homeownership, rurality, firearm ownership, alcohol consumption, military service, and history of non-traffic arrest. Q-values, also known as FDR-adjusted (or FDR-corrected) p-values, were calculated using the Benjamini-Yekutieli method [7]. Q-values represent the probability that the given difference would be a false discovery; they represent the expected proportion of "false positives" that would be seen among the collection of all differences whose q-values were at or below the given q-value. Item non-responses are not reported in the tables but are included in the prevalence calculations.

Table S9. Party/MAGA affiliation and personal willingness to engage in violence in support of or opposition to the federal government

How willing would you personally be	**Population Estimates by Party Category for 2025***							
	**MAGA Affiliation**						**No MAGA Affiliation**	
	**MAGA Republican (n = 1536)**		**MAGA Supporter, Republican (n = 694)**		**MAGA Supporter, Non-Republican (n = 194)**		**Non-MAGA, strong Republican (n = 304)**	
	**Unweighted n**	**Weighted % (95% CI)**	**Unweighted n**	**Weighted % (95% CI)**	**Unweighted n**	**Weighted % (95% CI)**	**Unweighted n**	**Weighted % (95% CI)**
In general, in a situation where you think force or violence is justified to advance an important political objective, how willing would you personally be to use force or violence this year…								
To support the government's enforcement of its policies								
Not asked the question†	170	11.9 (9.9, 14.3)	84	14.0 (10.9, 17.9)	27	15.5 (9.2, 24.8)	57	19.3 (13.8, 26.4)
Not willing	1123	73.0 (69.9, 75.9)	514	72.2 (67.4, 76.6)	121	55.7 (45.3, 65.6)	218	68.6 (60.4, 75.8)
Somewhat willing	182	10.9 (9.0, 13.1)	83	11.5 (8.7, 15.1)	24	8.9 (5.2, 14.9)	23	8.6 (4.8, 15.2)
Very or completely willing	38	2.4 (1.5, 3.8)	9	1.4 (0.7, 3.0)	17	17.0 (9.8, 27.7)	5	3.2 (0.8, 11.2)
Non-response	19	*1.8 (1.0, 3.1)*	4	0.8 (0.2, 2.8)	4	2.9 (0.7, 11.1)	1	0.3 (0.0, 2.0)
aPD; q-value‡	2.02 (0.41, 3.63); 0.18		0.69 (-0.87, 2.26); 1.00		13.68 (5.17, 22.2); 0.03		2.67 (-1.74, 7.08); 1.00	
To oppose the government's enforcement of its policies								
Not asked the question†	170	11.9 (9.9, 14.3)	84	14.0 (10.9, 17.9)	27	15.5 (9.2, 24.8)	57	19.3 (13.8, 26.4)
Not willing	1209	77.1 (74.1, 79.9)	547	75.7 (70.9, 80.0)	121	53.2 (42.9, 63.2)	221	70.5 (62.5, 77.4)
Somewhat willing	111	6.9 (5.4, 8.7)	56	8.6 (5.9, 12.3)	24	12.8 (7.5, 21.0)	19	7.7 (3.9, 14.6)
Very or completely willing	23	2.3 (1.4, 4.0)	3	0.8 (0.2, 2.8)	17	15.6 (8.8, 26.0)	7	2.5 (0.8, 7.0)
Non-response	19	1.8 (1.0, 3.1)	4	0.8 (0.2, 2.8)	4	2.9 (0.7, 11.1)	0	0.0 (0.0, 0.0)
aPD; q-value‡	-0.03 (-2.08, 2.01); 1.00		-1.42 (-3.33, 0.5); 1.00		9.53 (1.64, 17.41); 0.22		-0.05 (-3.25, 3.14); 1.00	
In a situation where you think force or violence is justified to advance an important political objective, how willing would you personally be to use force or violence against a person because they are…								
A person who speaks out against government policies or the President								
Not asked the question†	170	11.9 (9.9, 14.3)	84	14.0 (10.9, 17.9)	27	15.5 (9.2, 24.8)	57	19.3 (13.8, 26.4)
Not willing	1244	78.9 (75.9, 81.6)	577	80.1 (75.6, 83.9)	135	60.4 (49.7, 70.2)	232	73.2 (65.1, 80.0)
Somewhat willing	82	5.8 (4.3, 7.7)	24	4.2 (2.4, 7.3)	12	7.5 (3.4, 15.7)	9	3.7 (1.2, 11.0)
Very or completely willing	21	1.8 (1.1, 3.2)	4	0.7 (0.2, 2.3)	14	12.4 (6.6, 22.1)	5	3.5 (1.2, 9.9)
Non-response	15	1.6 (0.8, 2.9)	5	1.0 (0.3, 3.0)	5	4.2 (1.1, 14.4)	1	0.2 (0.0, 1.5)
aPD; q-value‡	1.28 (-0.42, 2.97); 1.00		0.06 (-1.45, 1.56); 1.00		10.3 (2.27, 18.33); 0.16		2.63 (-1.41, 6.68); 1.00	
A person who joins demonstrations against government policies or the President								
Not asked the question†	170	11.9 (9.9, 14.3)	84	14.0 (10.9, 17.9)	27	15.5 (9.2, 24.8)	57	19.3 (13.8, 26.4)
Not willing	1190	75.9 (72.8, 78.7)	562	78.9 (74.5, 82.8)	132	58.9 (48.3, 68.8)	222	69.2 (61.1, 76.3)
Somewhat willing	128	7.9 (6.3, 10.0)	39	5.2 (3.4, 7.9)	17	10.5 (5.5, 19.4)	15	8.1 (4.0, 15.6)
Very or completely willing	27	2.6 (1.6, 4.2)	4	0.9 (0.3, 2.6)	12	10.9 (5.6, 20.1)	8	3.1 (1.1, 8.1)
Non-response	17	1.7 (0.9, 3.1)	5	1.0 (0.3, 3.0)	5	4.2 (1.1, 14.4)	2	0.3 (0.1, 1.3)
aPD; q-value‡	1.69 (-0.16, 3.53); 0.73		0.02 (-1.57, 1.61); 1.00		8.97 (1.49, 16.46); 0.22		1.97 (-1.32, 5.26); 1.00	

Table S9, continued

How willing would you personally be	Population Estimates by Party Category for 2025*							
	MAGA Affiliation						No MAGA Affiliation	
	MAGA Republican (n = 1536)		MAGA Supporter, Republican (n = 694)		MAGA Supporter, Non-Republican (n = 194)		Non-MAGA, strong Republican (n = 304)	
	Unweighted n	Weighted % (95% CI)	Unweighted n	Weighted % (95% CI)	Unweighted n	Weighted % (95% CI)	Unweighted n	Weighted % (95% CI)
A reporter or journalist whose stories are critical of government policies or the President	0	0.0 (0.0, 0.0)	0	0.0 (0.0, 0.0)	0	0.0 (0.0, 0.0)	0	0.0 (0.0, 0.0)
Not asked the question†	170	11.9 (9.9, 14.3)	84	14.0 (10.9, 17.9)	27	15.5 (9.2, 24.8)	57	19.3 (13.8, 26.4)
Not willing	1240	78.8 (75.9, 81.5)	569	78.6 (74.0, 82.6)	132	58.5 (47.9, 68.4)	230	72.6 (64.5, 79.4)
Somewhat willing	79	5.3 (4.0, 7.0)	28	5.0 (3.0, 8.1)	12	6.5 (2.8, 14.5)	11	4.0 (1.9, 8.4)
Very or completely willing	27	2.3 (1.4, 3.8)	7	1.4 (0.6, 3.3)	17	15.3 (8.9, 25.2)	4	3.8 (1.0, 12.9)
Non-response	16	1.6 (0.9, 3.0)	6	1.0 (0.4, 3.0)	5	4.2 (1.1, 14.4)	2	0.3 (0.1, 1.3)
aPD; q-value‡	2.2 (0.53, 3.88); 0.14		1.17 (-0.56, 2.89); 1.00		11.89 (4.11, 19.66); 0.05		3.46 (-1.38, 8.3); 1.00	
This question turns things around: in a situation where you think force or violence is justified to advance an important political objective, how willing would you personally be to use force or violence against a person because they are…								
A person who speaks out in favor of government policies or the President								
Not asked the question†	170	11.9 (9.9, 14.3)	84	14.0 (10.9, 17.9)	27	15.5 (9.2, 24.8)	57	19.3 (13.8, 26.4)
Not willing	1281	81.4 (78.5, 83.9)	586	81.8 (77.5, 85.4)	138	64.2 (53.8, 73.5)	228	71.5 (63.5, 78.4)
Somewhat willing	43	3.3 (2.3, 4.9)	13	1.8 (1.0, 3.3)	11	6.5 (3.0, 13.2)	10	5.5 (2.6, 11.5)
Very or completely willing	23	1.8 (1.0, 3.0)	5	1.2 (0.4, 3.9)	13	10.9 (5.8, 19.6)	7	3.1 (0.8, 11.1)
Non-response	15	1.6 (0.9, 3.0)	6	1.2 (0.4, 3.1)	4	2.9 (0.7, 11.1)	2	0.5 (0.1, 2.0)
aPD; q-value‡	0.62 (-0.98, 2.22); 1.00		0.05 (-1.82, 1.92); 1.00		8.6 (1.37, 15.83); 0.23		1.97 (-2.34, 6.27); 1.00	
A person who joins demonstrations in favor of government policies or the President								
Not asked the question†	170	11.9 (9.9, 14.3)	84	14.0 (10.9, 17.9)	27	15.5 (9.2, 24.8)	57	19.3 (13.8, 26.4)
Not willing	1284	81.3 (78.4, 83.9)	585	82.1 (77.9, 85.6)	136	58.9 (48.2, 68.7)	229	71.3 (63.2, 78.3)
Somewhat willing	44	3.2 (2.2, 4.8)	15	1.9 (1.1, 3.4)	10	10.0 (4.7, 19.8)	10	6.0 (2.9, 12.1)
Very or completely willing	18	1.8 (1.0, 3.3)	4	0.8 (0.2, 2.5)	15	12.7 (7.0, 21.9)	6	2.9 (0.7, 11.3)
Non-response	16	1.7 (1.0, 3.1)	6	1.2 (0.4, 3.1)	5	3.0 (0.8, 11.0)	2	0.5 (0.1, 2.0)
aPD; q-value‡	0.82 (-0.81, 2.44); 1.00		-0.32 (-1.82, 1.17); 1.00		10.29 (2.57, 18); 0.13		1.82 (-2.4, 6.04); 1.00	
A reporter or journalist whose stories support government policies or the President								
Not asked the question†	170	11.9 (9.9, 14.3)	84	14.0 (10.9, 17.9)	27	15.5 (9.2, 24.8)	57	19.3 (13.8, 26.4)
Not willing	1290	81.4 (78.5, 84.0)	583	81.9 (77.7, 85.4)	139	63.7 (53.1, 73.1)	229	72.2 (64.3, 79.0)
Somewhat willing	36	2.9 (1.9, 4.5)	15	2.0 (1.1, 3.6)	10	6.1 (2.4, 14.4)	11	5.0 (2.4, 10.0)
Very or completely willing	22	2.2 (1.3, 3.7)	6	0.9 (0.3, 2.6)	13	11.8 (6.4, 20.7)	6	3.3 (0.9, 11.1)
Non-response	14	1.6 (0.9, 2.9)	6	1.2 (0.4, 3.1)	4	2.9 (0.7, 11.1)	1	0.2 (0.0, 1.5)
aPD; q-value‡	0.32 (-1.49, 2.14); 1.00		-1.14 (-2.76, 0.48); 1.00		5.95 (-0.48, 12.38); 0.70		1.42 (-2.87, 5.72); 1.00	

Table S9, continued.

How willing would you personally be	Population Estimates by Party Category for 2025*							
	No MAGA Affiliation							
	Non-MAGA, leans or not strong Republican (n = 1003)		Non-MAGA, Independent (n = 960)		Non-MAGA, leans or not strong Democrat (n = 1940)		Non-MAGA, strong Democrat (n = 1405)	
	Unweighted n	Weighted % (95% CI)	Unweighted n	Weighted % (95% CI)	Unweighted n	Weighted % (95% CI)	Unweighted n	Weighted % (95% CI)
In general, in a situation where you think force or violence is justified to advance an important political objective, how willing would you personally be to use force or violence this year…								
To support the government's enforcement of its policies								
Not asked the question†	210	26.3 (22.6, 30.3)	241	28.0 (24.3, 32.1)	451	23.4 (21.0, 26.0)	372	25.5 (22.7, 28.6)
Not willing	700	65.6 (61.4, 69.6)	629	62.7 (58.4, 66.7)	1380	70.1 (67.3, 72.8)	958	68.7 (65.4, 71.8)
Somewhat willing	76	6.0 (4.3, 8.4)	60	6.2 (4.4, 8.7)	75	4.2 (3.1, 5.7)	54	4.2 (2.8, 6.1)
Very or completely willing	9	1.0 (0.5, 2.1)	16	1.9 (1.0, 3.5)	17	1.3 (0.7, 2.4)	11	1.3 (0.6, 2.9)
Non-response	6	1.1 (0.4, 3.3)	10	1.2 (0.6, 2.7)	13	1.0 (0.5, 2.0)	6	0.3 (0.1, 0.7)
aPD; q-value‡	0.52 (-0.89, 1.92); 1.00		0.01 (-1.57, 1.59); 1.00		-0.2 (-1.58, 1.18); 1.00		Reference	
To oppose the government's enforcement of its policies								
Not asked the question†	210	26.3 (22.6, 30.3)	241	28.0 (24.3, 32.1)	451	23.4 (21.0, 26.0)	372	25.5 (22.7, 28.6)
Not willing	710	65.2 (61.0, 69.2)	584	57.9 (53.7, 62.1)	1196	61.4 (58.5, 64.3)	824	58.4 (54.9, 61.8)
Somewhat willing	64	5.5 (3.8, 7.9)	98	10.3 (8.0, 13.3)	243	11.9 (10.1, 14.0)	173	12.4 (10.2, 15.0)
Very or completely willing	13	2.0 (1.0, 3.8)	20	2.1 (1.2, 3.6)	32	2.2 (1.4, 3.4)	27	*3.4 (2.2, 5.3)*
Non-response	4	1.0 (0.3, 3.3)	13	1.6 (0.8, 3.2)	14	1.0 (0.5, 2.0)	5	0.2 (0.1, 0.7)
aPD; q-value‡	-0.51 (-2.67, 1.65); 1.00		-1.91 (-3.95, 0.12); 0.67		-1.44 (-3.32, 0.45); 1.00		Reference	
In a situation where you think force or violence is justified to advance an important political objective, how willing would you personally be to use force or violence against a person because they are…								
A person who speaks out against government policies or the President								
Not asked the question†	210	26.3 (22.6, 30.3)	241	28.0 (24.3, 32.1)	451	23.4 (21.0, 26.0)	372	25.5 (22.7, 28.6)
Not willing	747	68.4 (64.2, 72.3)	671	64.6 (60.4, 68.7)	1427	72.4 (69.6, 74.9)	991	70.6 (67.3, 73.6)
Somewhat willing	34	3.8 (2.4, 6.0)	22	3.7 (2.3, 6.1)	33	2.2 (1.4, 3.4)	17	1.5 (0.8, 2.7)
Very or completely willing	7	0.8 (0.3, 1.9)	16	2.7 (1.5, 4.8)	16	1.4 (0.7, 2.5)	17	2.2 (1.2, 4.0)
Non-response	3	0.7 (0.2, 3.1)	6	0.9 (0.3, 2.4)	9	0.7 (0.3, 1.6)	4	0.2 (0.1, 0.7)
aPD; q-value‡	-0.1 (-1.66, 1.45); 1.00		0.17 (-1.88, 2.23); 1.00		-0.86 (-2.36, 0.65); 1.00		Reference	
A person who joins demonstrations against government policies or the President								
Not asked the question†	210	26.3 (22.6, 30.3)	241	28.0 (24.3, 32.1)	451	23.4 (21.0, 26.0)	372	25.5 (22.7, 28.6)
Not willing	747	68.2 (64.0, 72.1)	671	64.9 (60.6, 68.9)	1420	72.1 (69.4, 74.7)	984	69.7 (66.5, 72.8)
Somewhat willing	32	3.8 (2.3, 6.2)	27	4.4 (2.8, 7.0)	40	2.7 (1.8, 4.0)	25	2.3 (1.4, 3.9)
Very or completely willing	7	0.8 (0.3, 1.8)	11	1.8 (0.8, 3.6)	15	1.1 (0.6, 1.9)	15	2.1 (1.2, 3.8)
Non-response	5	0.9 (0.3, 3.0)	6	0.9 (0.3, 2.4)	10	0.7 (0.3, 1.6)	5	0.3 (0.1, 0.7)
aPD; q-value‡	-0.38 (-1.86, 1.09); 1.00		-0.77 (-2.6, 1.05); 1.00		-1.15 (-2.51, 0.21); 0.94		Reference	

Table S9, continued.

How willing would you personally be	Population Estimates by Party Category for 2025*							
	No MAGA Affiliation							
	Non-MAGA, leans or not strong Republican (n = 1003)		Non-MAGA, Independent (n = 960)		Non-MAGA, leans or not strong Democrat (n = 1940)		Non-MAGA, strong Democrat (n = 1405)	
	Unweighted n	Weighted % (95% CI)	Unweighted n	Weighted % (95% CI)	Unweighted n	Weighted % (95% CI)	Unweighted n	Weighted % (95% CI)
A reporter or journalist whose stories are critical of government policies or the President	0	0.0 (0.0, 0.0)	0	0.0 (0.0, 0.0)	0	0.0 (0.0, 0.0)	0	0.0 (0.0, 0.0)
Not asked the question†	210	26.3 (22.6, 30.3)	241	28.0 (24.3, 32.1)	451	23.4 (21.0, 26.0)	372	25.5 (22.7, 28.6)
Not willing	751	68.7 (64.5, 72.7)	675	65.8 (61.6, 69.8)	1432	72.7 (70.0, 75.2)	990	70.4 (67.2, 73.4)
Somewhat willing	30	3.3 (1.9, 5.6)	20	3.1 (1.9, 5.2)	25	1.7 (1.1, 2.9)	25	2.7 (1.7, 4.4)
Very or completely willing	6	0.8 (0.4, 2.0)	13	2.0 (1.0, 4.0)	16	1.3 (0.7, 2.3)	11	1.1 (0.5, 2.2)
Non-response	4	0.8 (0.2, 3.1)	7	1.0 (0.4, 2.5)	12	0.9 (0.4, 1.8)	3	0.3 (0.1, 1.0)
aPD; q-value‡	0.55 (-0.84, 1.93); 1.00		0.36 (-1.35, 2.07); 1.00		-0.22 (-1.33, 0.88); 1.00		Reference	
This question turns things around: in a situation where you think force or violence is justified to advance an important political objective, how willing would you personally be to use force or violence against a person because they are…								
A person who speaks out in favor of government policies or the President								
Not asked the question†	210	26.3 (22.6, 30.3)	241	28.0 (24.3, 32.1)	451	23.4 (21.0, 26.0)	372	25.5 (22.7, 28.6)
Not willing	756	68.9 (64.7, 72.9)	658	63.7 (59.5, 67.8)	1386	69.6 (66.8, 72.3)	953	67.4 (64.0, 70.5)
Somewhat willing	22	2.6 (1.4, 4.9)	32	4.7 (3.1, 7.1)	71	4.7 (3.5, 6.3)	50	4.5 (3.1, 6.5)
Very or completely willing	7	1.0 (0.4, 2.1)	18	2.5 (1.4, 4.3)	17	1.4 (0.8, 2.6)	17	2.0 (1.1, 3.4)
Non-response	6	1.2 (0.4, 3.3)	7	1.1 (0.4, 2.5)	11	0.9 (0.4, 1.8)	9	0.6 (0.3, 1.3)
aPD; q-value‡	-0.18 (-1.65, 1.29); 1.00		0.09 (-1.69, 1.88); 1.00		-0.46 (-1.86, 0.95); 1.00		Reference	
A person who joins demonstrations in favor of government policies or the President								
Not asked the question†	210	26.3 (22.6, 30.3)	241	28.0 (24.3, 32.1)	451	23.4 (21.0, 26.0)	372	25.5 (22.7, 28.6)
Not willing	760	69.2 (65.0, 73.2)	658	64.4 (60.1, 68.4)	1381	69.7 (66.9, 72.4)	945	66.6 (63.2, 69.8)
Somewhat willing	22	2.8 (1.5, 5.1)	28	3.7 (2.3, 5.8)	78	5.3 (4.0, 7.0)	58	5.3 (3.8, 7.5)
Very or completely willing	4	0.5 (0.2, 1.6)	21	2.8 (1.7, 4.7)	14	0.7 (0.4, 1.3)	16	1.9 (1.0, 3.3)
Non-response	5	1.1 (0.4, 3.3)	8	1.1 (0.5, 2.6)	12	0.8 (0.4, 1.8)	10	0.7 (0.3, 1.5)
aPD; q-value‡	-0.51 (-1.83, 0.81); 1.00		0.47 (-1.3, 2.24); 1.00		-1.11 (-2.27, 0.06); 0.66		Reference	
A reporter or journalist whose stories support government policies or the President								
Not asked the question†	210	26.3 (22.6, 30.3)	241	28.0 (24.3, 32.1)	451	23.4 (21.0, 26.0)	372	25.5 (22.7, 28.6)
Not willing	761	69.5 (65.3, 73.4)	667	65.5 (61.3, 69.5)	1407	70.9 (68.1, 73.5)	952	66.7 (63.3, 69.9)
Somewhat willing	21	2.2 (1.2, 3.8)	29	3.9 (2.5, 6.1)	53	3.8 (2.7, 5.2)	48	4.9 (3.4, 6.9)
Very or completely willing	4	0.9 (0.2, 3.7)	11	1.4 (0.7, 2.8)	14	1.1 (0.6, 2.3)	21	2.4 (1.4, 4.0)
Non-response	5	1.1 (0.4, 3.3)	8	1.1 (0.5, 2.6)	11	0.8 (0.4, 1.7)	8	0.5 (0.2, 1.3)
aPD; q-value‡	-1.1 (-2.81, 0.61); 1.00		-2.04 (-3.72, -0.37); 0.21		-1.93 (-3.44, -0.42); 0.17		Reference	

* Questions were asked of all participants.

† Respondents who did not answer the question "In general...to advance an important political objective that you support" (n = 57) or who answered “never justified” to all prior questions on the use of force or violence to advance political objectives were not asked questions on their personal willingness to use political violence.

‡ Adjusted prevalence differences (aPDs) are absolute percentage point (pp) differences for “very or completely willing” responses. They are adjusted for age, gender, race and ethnicity, education, income, Census division, marital status, homeownership, rurality, firearm ownership, alcohol consumption, military service, and history of non-traffic arrest. Q-values, also known as FDR-adjusted (or FDR-corrected) p-values, were calculated using the Benjamini-Yekutieli method [7]. Q-values represent the probability that the given difference would be a false discovery; they represent the expected proportion of “false positives” that would be seen among the collection of all differences whose q-values were at or below the given q-value. Item non-responses are not reported in the tables but are included in the prevalence calculations.

REFERENCES

1. Grinnell College National Poll. 52% of Americans believe democracy facing “major threat.” Study #2243. 2021 October 20. https://www.grinnell.edu/news/52-americans-believe-democracy-facing-major-threat.
2. NPR/PBS NewsHour/Marist National Poll. Trust in elections, threat to democracy, November 2021. 2021 November 1. https://maristpoll.marist.edu/polls/npr-pbs-newshour-marist-national-poll-trust-in-elections-threat-to-democracy-biden-approval-november-2021/.
3. The Economist/YouGov Poll. 2021 June 13-16. https://docs.cdn.yougov.com/uagnfc262c/econTabReport.pdf.
4. Democracy Fund Voter Study Group. Guide to views of The Electorate Research Survey. 2021 December. https://www.voterstudygroup.org/data/voter-survey.
5. Hart Research Associates. The New Republic Democracy Survey (Study #14230). 2022 March. https://newrepublic.com/article/166027/democracy-poll.
6. United States Census Bureau. National Population by Characteristics: 2020-2024. June 2025. https://www.census.gov/data/tables/time-series/demo/popest/2020s-national-detail.html.
7. Benjamini Y, Yekutieli D. The control of the false discovery rate in multiple testing under dependency. Ann Stat. 2001;29(4):1165-1188.
